# Functional dynamics of water in New Delhi metallo‐β‐lactamase catalysis

**DOI:** 10.1002/pro.70633

**Published:** 2026-05-18

**Authors:** Palanisamy Kandhan, Chuanye Xiong, Timothy Palzkill, Peng Tao

**Affiliations:** ^1^ Department of Chemistry, O'Donnell Data Science and Research Computing Institute, Center for Drug Discovery, Design, and Delivery (CD4) Southern Methodist University Dallas Texas USA; ^2^ Verna and Marrs McLean Department of Biochemistry and Molecular Pharmacology Baylor College of Medicine Houston Texas USA

**Keywords:** catalytic water, cefiderocol, molecular dynamics, NDM‐1, solvent dynamics

## Abstract

Water plays a critical role in the hydrolysis of antibiotics by New Delhi metallo‐β‐lactamases (NDMs). The reaction proceeds through a nucleophilic attack on the β‐lactam ring, followed by cleavage of the C–N bond and formation of an anionic nitrogen intermediate. This intermediate is then protonated by a water molecule diffusing from the bulk solvent. Although the catalytic mechanism of NDMs has been extensively studied, the molecular mechanism governing water entry into the active site during hydrolysis remains poorly understood. Here, we use molecular dynamics simulations to characterize the dynamics of catalytic water molecules during NDM‐mediated hydrolysis. We examine the NDM‐1, substrate‐bound, intermediate, and product states of the NDM‐1–cefiderocol system. In the enzyme‐intermediate state, conformational rearrangements of loops L3 and L10, together with cefiderocol coordination to Zn2, expand the active site and increase the Zn1–Zn2 separation to ~4.5 Å. These changes promote formation of a water channel toward Zn2, allowing water to enter the active site and protonate the anionic nitrogen, thereby completing hydrolysis. Concurrently, structural changes restrict the water‐entry pathway associated with Zn1. These findings reveal how local protein dynamics and water influx couple to catalysis, providing mechanistic insights into NDM function and informing strategies to inhibit these enzymes.

## 1 INTRODUCTION

β‐Lactam antibiotics remain a cornerstone of treatment for bacterial infections (Drawz & Bonomo, 2010; Fisher et al., 2005; Wright, 2011). However, their overuse has driven antimicrobial resistance (AMR), a global health threat linked to nearly five million deaths annually. A major contributor to AMR is the emergence of β‐lactamase‐producing multidrug‐resistant pathogens, which inactivate these antibiotics by hydrolyzing the β‐lactam ring. β‐Lactamases are categorized into four classes (A, B, C, and D). Classes A, C, and D are serine‐β‐lactamases (SBLs) that employ an active‐site serine as the nucleophile, whereas class B enzymes are metallo‐β‐lactamases (MBLs) that use active‐site‐bound zinc ions for catalysis. Defining their catalytic mechanism is essential not only for understanding how these enzymes inactivate antibiotics but also for guiding the design of effective inhibitors.

β‐Lactamases catalyze the hydrolysis of the β‐lactam ring (a four‐membered cyclic amide) in β‐lactam antibiotics, thereby inactivating them. This reaction proceeds in two steps: first, a nucleophilic group in the enzyme attacks the β‐lactam ring, forming an enzyme–intermediate (EI) complex; second, a catalytic water molecule hydrolyzes this intermediate to release the inactivated antibiotic. Although water plays an essential role in this process, its positioning and function vary among β‐lactamase (BL) classes.

In SBLs, the catalytic water molecule is generally preorganized but may undergo repositioning or activation during catalysis. For instance, in class A, it shifts toward Glu166 after acylation; in class C, it helps stabilize the transition state; and in class D, it is activated by a carbamylated lysine (Golemi et al., 2001; He et al., 2020; Lima & van der Kamp, 2025). While SBLs rely on a preorganized catalytic water, MBLs depend even more fundamentally on solvent‐derived water: recruited directly from bulk solvent, it drives both intermediate complex production and subsequent protonation (Tripathi & Nair, 2015). This unique reliance not only distinguishes MBLs mechanistically but also underscores their clinical significance.

MBLs pose a major threat to public health due to their unique zinc ion coordination, which enables hydrolysis of nearly all known β‐lactam antibiotics. They exhibit broad substrate specificity, and no clinically approved inhibitors are currently available. Of particular concern is New Delhi metallo‐β‐lactamase 1 (NDM‐1), a subclass B1 enzyme first identified in 2008 (Yong et al., 2009). Plasmid‐borne NDM‐1 has spread globally, conferring resistance to nearly all β‐lactams and posing significant clinical challenges (Mojica et al., 2022; Yang & Crowder, 2015). Since its discovery, NDM‐1 has evolved into more than 40 variants, further amplifying the problem of antibiotic resistance (Farhat & Khan, 2020; Tran et al., 2022).

The canonical crystal structure of NDM‐1 reveals two zinc ions (Zn^2+^) in the active site, designated Zn1 and Zn2. Zn1 is coordinated by His120, His122, His189, and a bridging water/hydroxide ion (W1), while Zn2 is coordinated by Asp124, Cys208, His250, along with W1 and an additional bound water molecule (W2) (Schenk et al., 2012; Yang et al., 2012). In one reported structure, Zn2 adopts an octahedral coordination geometry, binding three water molecules in addition to the protein residues (Green et al., 2011). Other studies suggest that Zn2 may also bind an extra water molecule, adopting a trigonal bipyramidal geometry with W2 to form a five‐coordinate complex (Guo et al., 2011; Kim et al., 2011; Simona et al., 2009). Although the presence of this additional water at the Zn2 site remains under debate, it has been proposed to contribute to catalysis (Dal Peraro et al., 2007; Suárez et al., 2002; Zhu et al., 2013).

The Zn1–Zn2 distance (~3.5 Å) is maintained within a shallow active‐site groove flanked by two flexible loops that regulate water access and substrate binding. Loop 3 (L3; residues 65–73) forms a hydrophobic pocket for substrate binding, while Loop 10 (L10; residues 210–230) contains Cys208, which coordinates Zn2, as well as Lys211 and Asn220, both of which interact with the substrate's carboxyl group (Drawz & Bonomo, 2010).

**FIGURE 1:**
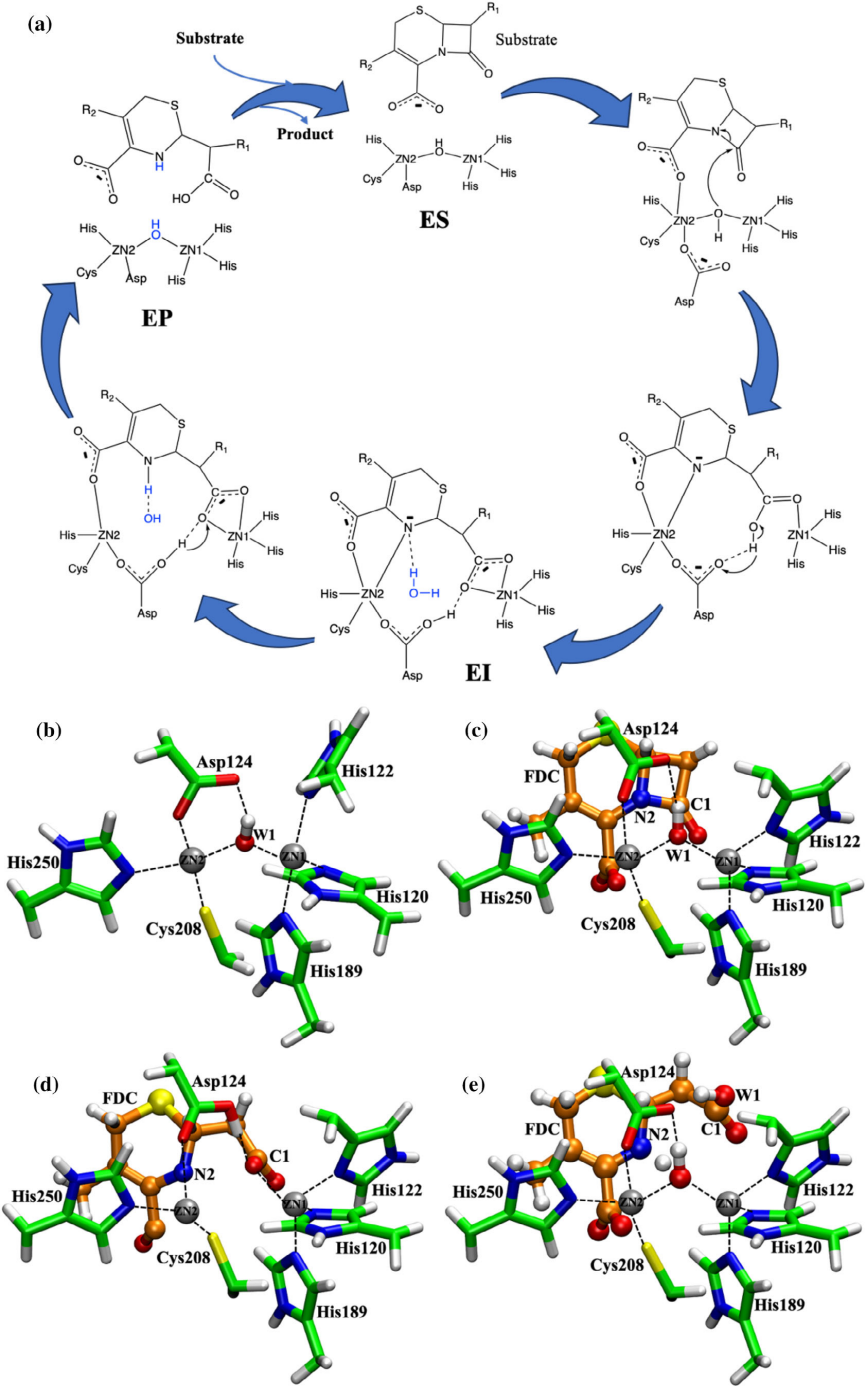
Catalytic mechanism of New Delhi metallo‐β‐lactamase 1 with cefiderocol (FDC). (a) Schematic representation of the catalytic cycle. (b–e) QM/MM geometry optimized structures of distinct catalytic states: (b) NDM‐1, (c) enzyme–substrate (ES) complex, (d) enzyme–intermediate (EI) complex, and (e) enzyme–product (EP) complex. Active site residues (side chains) are shown as green licorice representations, the cephalosporin moiety of FDC is shown as an orange ball‐and‐stick model, and zinc ions (Zn1 and Zn2) are shown as black spheres. Key interactions, including coordination and hydrogen bonds, are indicated by dotted lines.

The catalytic water molecule coordinated to the zinc ion plays a key role in proton transfer following intermediate formation (J. Jitonnom et al., 2017; W. Jitonnom, Oláh, et al., 2025; Meelua, Oláh, et al., 2022). NDM‐1 catalysis proceeds through a series of water‐mediated events (Figure 1a). In the enzyme‐substrate (ES) state, the bridging water molecule W1, coordinated between Zn1 and Zn2, initiates cleavage of the β‐lactam ring C–N bond, leading to the formation of the enzyme‐intermediate (EI) complex. Zn1 and Zn2 stabilize the newly formed carboxylate oxygen and the anionic nitrogen of the β‐lactam. Protonation of the anionic nitrogen occurs either via W2 or a bulk solvent water molecule, ultimately generating the hydrolyzed enzyme–product (EP) complex (Krivitskaya & Khrenova, 2022a, 2022b; Sun et al., 2018; Xu et al., 2007). A charged Asp124, in the absence of W2 at the Zn2 site, promotes C–N bond cleavage and EI complex formation, consistent with spectroscopic studies of NDM‐1 (Yang et al., 2012; Zhu et al., 2013). In contrast, when W2 is present, Asp124 does not initiate C–N bond cleavage. Previous mechanistic studies of NDM‐1 have suggested that protonation of the anionic nitrogen occurs via a bulk water molecule, rather than from W1 or Asp124 (Tripathi & Nair, 2015). In addition, hydrolysis of NDM‐1–ampicillin has been shown to involve proton transfer as the rate‐determining step, with protonation mediated by a solvent water (Lai & Li, 2025). Nuclear magnetic resonance (NMR) and crystallographic data further corroborate the role of bulk solvent in protonating the intermediate (Feng et al., 2017; Lisa et al., 2017). These studies indicate that bulk water molecules access the active site during the EI state to facilitate protonation. However, the underlying water dynamics and pathways of water entry remain enigmatic.

Cefiderocol (FDC), a siderophore cephalosporin considered a “last‐resort” antibiotic for multidrug‐resistant infections, is efficiently hydrolyzed by NDM‐1. In contrast, other MBLs, such as VIM‐2 and IMP‐1, show reduced catalytic activity against FDC (Seifert et al., 2023; Takemura et al., 2023; Viale et al., 2023). A recent study suggested that an extensive interaction network between FDC and the active site underlies these differences in activity (Warecki et al., 2025). However, the mechanistic basis of this interaction network, particularly the often‐overlooked role of water dynamics within MBL active sites, remains poorly understood, especially in the context of clinically important antibiotics and high‐risk MBLs such as NDMs.

Here, we employ multiscale simulations to investigate the role of water in orchestrating NDM‐1 catalysis, using FDC as a model substrate. We examined how water accesses the NDM‐1 active site during the EI complex formation and how conformational gating regulates this process. By integrating hybrid quantum mechanics/molecular mechanics (QM/MM) simulations with extensive sampling across the NDM‐1, ES, EI, and EP complexes (Figure 1b to 1e, respectively), combined with the Markov state model (MSM), we elucidate how the dynamic water network enables completion of FDC hydrolysis by NDM‐1.

## 2 RESULTS AND DISCUSSION

### 2.1 Role of the catalytic water in NDM‐1 hydrolysis

The crystal structure (PDB ID: 1ZNB) of NDM‐1 reveals two water molecules in the active site: W1, bridging Zn1 and Zn2, and W2, coordinating with Zn2 along with Asp124, Cys208, and His250 (Figure S1B) (Yang et al., 2014). In contrast, some crystal structures (PDB ID: 5ZGZ) lack the W2 water molecule in the active site. To evaluate its catalytic relevance, we performed QM/MM geometry optimizations (see Methods) of the NDM‐1–FDC complex in the presence and absence of W2, respectively. In the model including W2, it is positioned between Zn2 and the O6 atom of the FDC cephem core carboxylate, forming a hydrogen bond (Figure S1C). Previous work has shown that the presence of W2 increases the energy barrier for forming a carbon–oxygen bond between FDC and W1, thereby inhibiting hydrolysis (Zhu et al., 2013). Consistent with this, our study also confirms that W2 sterically hinders direct coordination between the FDC cephem core carboxylate and Zn2, an interaction observed experimentally in the EI complex (Yang et al., 2012). These findings suggest that crystallographic W2 does not contribute to catalysis and may impede hydrolysis. Instead, the catalytic water appears to originate from bulk solvent. We therefore excluded W2 from subsequent modeling to better characterize the identity and dynamics of the true catalytic water molecule.

### 2.2 QM/MM analysis of the catalytic mechanism of NDM‐1 against cefiderocol

We began with an ES complex conformation without the W2 water molecule, which was optimized using the SCC‐DFTB/CHARMM method. To validate our QM/MM optimized ES structure, key interactions were compared with previously published QM/MM MD data (Tripathi & Nair, 2015) (Table S1), showing good agreement. Based on this validated ES conformation, the EI and EP complexes were subsequently generated and optimized (see Methods). In the ES complex, the oxygen atom of W1 (OW1) is positioned 3.06 Å from the β‐lactam carbonyl carbon (C1) of FDC (Figure 1c) and forms a hydrogen bond with Asp124.

In the first step, OW1 attacks C1 of FDC, leading to cleavage of the C1–N2 bond and formation of an anionic intermediate (N2^−^). This reaction converts the amide carbonyl into a carboxyl group (ring‐opened β‐lactam carboxyl). The proton from this newly formed group is subsequently transferred to the adjacent residue Asp124, converting it from its anionic to neutral form. As a result, the ring‐opened β‐lactam carboxyl group becomes deprotonated, yielding a carboxylate (COO^−^). We validated the QM/MM optimized EI structure by comparison with the crystal structure of NDM‐1–cefuroxime (PDB ID: 5O2E) (Table 1), showing close agreement with experimental observations (Feng et al., 2014; Raczynska et al., 2018). However, two notable discrepancies are observed in Table 1 for the Zn2⋯Oδ1(Asp124) and Zn1⋯OW1(FDC) distances. For the Zn2⋯Oδ1(Asp124) distance, Asp124 is modeled in its neutral (protonated) form following proton transfer. As a result, it no longer strongly coordinates Zn2 and instead forms a hydrogen bond with OW1 of the newly formed carboxylate group (Figure 1d). In contrast, in the corresponding crystal structure, Asp124 is typically negatively charged and maintains stronger coordination with Zn2. For the Zn1⋯OW1(FDC) distance, the β‐lactam carbonyl of FDC is converted to a ring‐opened carboxylate group. This negatively charged (OW1/O3) group coordinates more strongly with Zn1, resulting in a shorter Zn1⋯OW1 distance. This behavior is consistent with the crystal structure of the NDM‐1–meropenem complex (PDB ID: 4EYL), which shows a comparable Zn1⋯OW1 distance (~2.24 Å).

**TABLE 1:** Comparison of key distances (Å) between NDM‐1–FDC complex in EI state based on the QM/MM calculations in this study and the crystal structure of NDM‐1–cefuroxime (PDB ID: 5O2E).

Distance pairs	NDM‐1–FDC (Å)	NDM‐1–cefuroxime (Å)
Zn1⋯Zn2	3.86	3.83
Zn1⋯Nε2(His120)	2.06	2.07
Zn1⋯Nδ1(His122)	2.06	2.00
Zn1⋯Nε2(His189)	2.04	1.98
Zn2⋯Sγ(Cys208)	2.40	2.34
Zn2⋯Nε2(His250)	2.17	2.12
Zn2⋯Oδ1(Asp124)	2.53	2.01
Zn2⋯N2(FDC)	2.10	2.35
Zn1⋯OW1(FDC)	2.06	2.87
Zn2⋯O6(FDC)	2.05	2.15
N(Asn220)⋯O7(FDC)	3.04	2.99
Nδ2(Asn220)⋯O3(FDC)	2.85	2.96
C1(FDC)⋯N2 (FDC)	2.64	2.46

In the second step, a bulk water molecule enters the active site and protonates the anionic nitrogen (N2^−^). The resulting hydroxide ion replaces W1 in the bridging position between the two zinc ions, while Asp124 transfers its proton back to the ring‐opened β‐lactam carboxylate (OW1) restoring its negative charge. Unlike the crystallographic W2 water, which inhibits EI formation, the bulk water molecule is essential for protonation and enters the active site following conformational changes. Protonation of N2^−^ is likely the rate‐limiting step in NDM‐1–FDC hydrolysis (Lai & Li, 2025; Liang et al., 2011; Linciano et al., 2019).

To investigate the forces governing bulk water entry and the associated conformational changes in the NDM‐1 active site, we analyzed the catalytic‐state dynamics, focusing on the water network and structural rearrangements.

### 2.3 Structural stability and dynamics of distinct NDM‐1 states

We examined the structural stability and dynamic behavior of the NDM‐1–FDC complex across its distinct catalytic states: NDM‐1, ES, EI, and EP. The NDM‐1 binding pocket is primarily hydrophobic, with Lys211, Asp212, and Asn220 contributing local polar interactions. To evaluate the flexibility and stability of the modeled complexes, we calculated backbone root mean square deviation (RMSD) and side chain root mean square fluctuation (RMSF). In all states, NDM‐1 remained structurally stable in complex with FDC, as indicated by average RMSD values below 2 Å (Figure S2 and Table S2).

To understand the conformational dynamics of NDM‐1, we assessed the flexibility of its structural regions and found that the overall fold remains consistently rigid across all four states (Figure S3). However, loops L3 and L10, along with the segment spanning residues 170–176, exhibit notable flexibility. Because L3 and L10 are part of the active site and participate in substrate binding, we focused our analysis on these regions. The 170–176 segment was excluded for clarity, as it lies distal to the active site and does not directly contribute to catalysis.

We next analyzed the hydrogen‐bonding interactions between FDC and NDM‐1. The number of hydrogen bonds and their occupancy percentages were calculated, and residues with higher occupancy (>50%), along with their average distances, are presented in Figure S4 and Table S3. In simulations of the ES complex, FDC consistently forms two hydrogen bonds with Asn220: one between the β‐lactam carbonyl (O3) of FDC and the side‐chain NH_2_ of Asn220 (O3⋯NH_2_), and the other between the FDC cephem core carboxylate group (O6) and the backbone NH of Asn220 (O6⋯NH) (Figure S5A). The catechol hydroxyl groups of FDC form two hydrogen bonds with Asp212: one with the side‐chain carboxylate (OH⋯O) and one with the backbone NH (OH⋯NH). In addition to these stable interactions, FDC forms transient, low‐occupancy hydrogen bonds with Asp43, Ala74, Gln123, Cys208, Ile210, Asp212, Ser217, Leu218, Gly129, Asp223, and Ser251. Hydrophobic interactions with residues in L3 and L10 further contribute to FDC stabilization.

In contrast, in the EI complex, FDC engages in more extensive hydrogen bonding (Figure S4D). The FDC ring‐opened β‐lactam carboxylate group forms a hydrogen bond with neutral Asp124 (O⋯HO), drawing Gln123 closer and enabling its backbone NH to interact with the FDC carbonyl (NH⋯O). The catechol hydroxyl groups form hydrogen bonds with the backbone carbonyl of Ile210 (OH⋯O) and the side‐chain hydroxyl of Ser251 (OH⋯O), while the backbone NH of His250 interacts with one of the catechol hydroxyls (NH⋯O). Additionally, the FDC carbonyl group forms a hydrogen bond with the side‐chain NH_2_ of Lys211 (O⋯NH2) (Figure S5B). Notably, FDC maintains two hydrogen bonds with Asn220 in the EI complex, mirroring those observed in the ES state. His122, Glu152, Gly207, and Asp223 also form low‐occupancy hydrogen bonds with FDC. Together, these conserved and transient interactions with active‐site residues may reorganize the water network and promote conformational changes in active‐site loops that facilitate catalysis.

### 2.4 Water distribution around the active site in the ES and EI states

**FIGURE 2:**
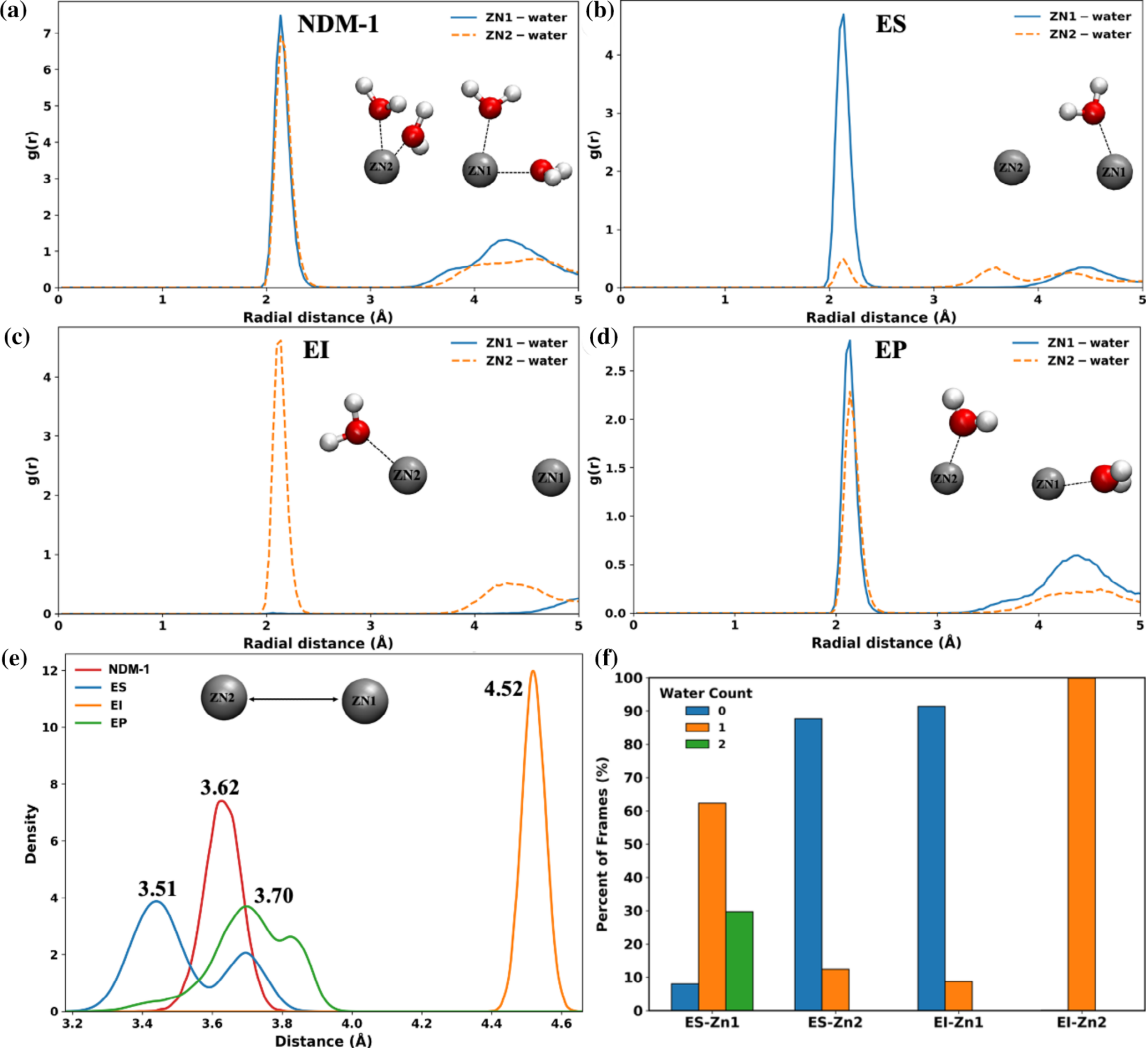
Radial distribution functions (RDFs) showing Zn–water interactions in distinct catalytic states of the NDM‐1–FDC complex: (a) NDM‐1, (b) ES, (c) EI, and (d) EP. (e) Zn1–Zn2 distance. (f) Number of water molecules within 3 Å of Zn1 and Zn2 in the ES and EI states, respectively.

To investigate differences in water distribution between the ES and EI states, we calculated radial distribution functions (RDFs) of water molecules around Zn1 and Zn2 (Figure 2). In both the NDM‐1 (Figure 2a) and EP (Figure 2d) states, Zn1 and Zn2 show similar distributions. In contrast, the ES state displays a clear asymmetry: a distinct water peak appears at 2.2 Å around Zn1, while Zn2 exhibits minimal water density (Figure 2b), likely due to steric hindrance from the FDC cephem core carboxylate group. In the EI state, a distinct water peak appears at 2.2 Å around Zn2, with negligible density near Zn1 (Figure 2c), indicating increased solvation of Zn2. This shift reflects Zn2 coordination to the anionic nitrogen (N2^−^) of FDC, which creates space for water entry. This hydration pattern differs from that of the ES state, where water is disfavored between the FDC cephem core carboxylate and Zn2, further supporting our earlier findings.

During catalysis, the Zn1−Zn2 distance increases from approximately 3.5 Å in the ES state to 4.5 Å in the EI state (Figure 2e). This increase results from N2^−^ coordination to Zn2, which creates space for additional water near Zn2 and facilitates proton transfer. Following protonation of N2^−^ in the EP state, the Zn1−Zn2 distance shortens to about 3.7 Å, consistent with the loss of N2^−^ coordination.

To gain insight into solvent behavior, we quantified the number of water molecules within 3 Å of Zn1 and Zn2 in ES and EI simulations (Figure 2f). In the ES state, 63% of simulations contain one water molecule near Zn1, whereas 30% contain two. In contrast, only 10% of simulations contain one water molecule near Zn2, while the remaining trajectories contain no water molecules within that distance. This pattern reverses in the EI state: 90% of frames lack water molecules near Zn1, whereas Zn2 is hydrated by a single water molecule in nearly every frame.

These results suggest that upon formation of the anionic intermediate, water coordination shifts from Zn1 to Zn2, enabling Zn2 to coordinate water for proton transfer. Zn2 thus functions as the primary catalytic center during hydrolysis by stabilizing the anionic intermediate and facilitating proton transfer, whereas Zn1 plays a secondary role due to its less stable water coordination.

### 2.5 Solvent accessible channels in the active site during NDM‐1 hydrolysis

**FIGURE 3:**
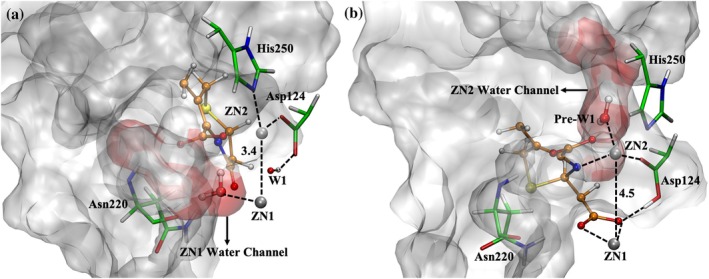
Active site rearrangements and water redistribution during FDC hydrolysis by NDM‐1. (a) Zn1 water channel in the ES state. FDC does not closely coordinate with either zinc ion. The Zn1–Zn2 distance is ~3.4 Å. (b) Zn2 water channel in the EI state. The Zn1 water channel disappears, and the Zn2 water channel emerges. The ring‐opened FDC intermediate coordinates strongly with both zinc ions, and its structural extension increases the Zn1–Zn2 distance to ~4.5 Å. Active site residues are shown as green licorice representations, the cephalosporin moiety of FDC is shown as an orange ball‐and‐stick representation, and zinc ions are shown as black spheres. The protein surface and water network are shown as surface representations. Key interactions, including coordination and hydrogen bonds, are indicated by dotted lines.

We found that the water network is consistently located near the zinc ions. An anchor water molecule was defined as one located within 3 Å of both Zn1 and Zn2. From this anchor water, neighboring water molecules within 6 Å and connected through hydrogen bonding interactions were identified to construct the local water network. The number of water molecules in this network was calculated over the simulations (Figure S6). This analysis revealed that a stable and persistent water network forms near Zn1 in the ES state and near Zn2 in the EI state, whereas other regions show more transient and less consistent water networks. Based on these observations, representative structures of the ES and EI states were selected to illustrate the water channels, as shown in Figure 3a,b.

In addition, we examined the shift in water coordination from Zn1 to Zn2 and its coupling to structural rearrangements within NDM‐1. These rearrangements involve Asp124 and FDC and coincide with the formation of the anionic intermediate. During the first step of hydrolysis, Asp124 abstracts a proton from the substrate ring‐opened β‐lactam carboxyl group, neutralizing its charge. In the ES state, Asp124 coordinates Zn2 and forms hydrogen bonds with W1 (Figure 3a). Upon formation of the EI state, Asp124 remains coordinated to Zn2 and forms a bridging interaction with Zn1 through the FDC ring‐opened β‐lactam carboxylate group (Figure 3b). This rearrangement positions Asp124 closer to Zn1. After hydrolysis is complete, Asp124 donates a proton back to the FDC ring‐opened β‐lactam carboxyl group and reestablishes interactions with Zn2 and W1.

Zn2 coordination to the anionic nitrogen of FDC in the EI state alters the geometry of the metal center. This interaction induces a conformational shift in FDC's cephalosporin moiety relative to its ES‐state structure. The carboxylate group generated upon β‐lactam ring opening also coordinates with Zn1 in the EI state. Together, these newly formed interactions couple FDC conformational changes with the active site geometry. Consequently, the extension of the FDC structure functions as a molecular switch, increasing the distance between the two zinc ions and thereby expanding the active site (Figure 3b). This expansion allows water molecules to access the Zn2 site through the gap between His250 and FDC.

**FIGURE 4:**
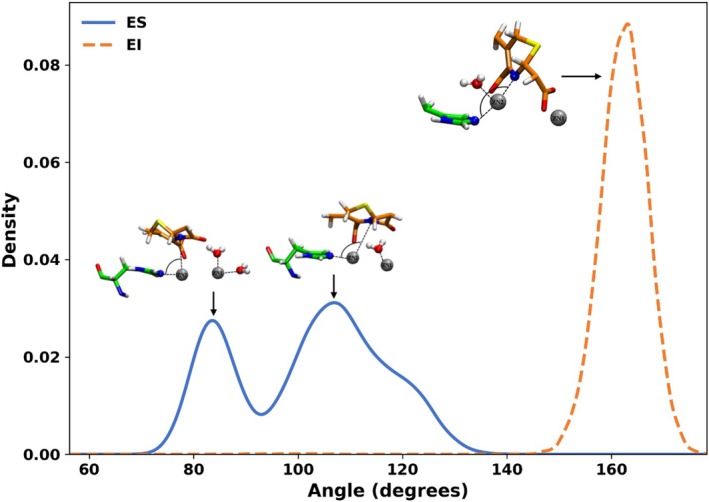
Distributions of the His250–Zn2–FDC angle (Nε2–Zn2–N2) in the ES and EI states. In the ES state, two populations are observed: a peak near 80°, corresponding to Zn1 coordination by two water molecules, and a peak near 105°, corresponding to Zn1 coordination by one water molecule. In the EI state, Zn1 no longer coordinates water, whereas Zn2 coordinates a single water molecule.

To examine active site rearrangements during hydrolysis, we plotted the His250–Zn2–FDC angle (Nε2–Zn2–N2) distribution for the ES and EI states (Figure 4). In the ES state, the distribution shows two distinct peaks at approximately 83° and 105°. The lower‐angle peak corresponds to a conformation in which Zn1 coordinates with two water molecules, while the higher‐angle peak corresponds to coordination with one. These differences are consistent with the water coordination patterns observed in Figure 2f. In the EI state, a dominant peak at 160° corresponds to the absence of water coordinated to Zn1. Moreover, 90% of EI‐state simulations lack water within coordination distance of Zn1, suggesting restricted access. These configurations represent a shift in water dynamics associated with catalysis, allowing a bulk water molecule to enter the Zn2 site through the spatial gap between His250 and FDC in the EI state.

The β‐lactam carbonyl oxygen (O3) in the ES state and newly formed carboxylate group in the EI state consistently form hydrogen bonds with Asn220 (Figure S4C,D). These observations suggest that Asn220, a residue on loop L10, functions as a gatekeeper that regulates water access to the active site. In the ES state, water can reach Zn1 through a channel within the gap adjacent to Asn220 and is stabilized by hydrogen bonds to both the β‐lactam carbonyl oxygen (O3) and FDC cephem core carboxylate (O6). This channel is referred to as the Zn1 water channel as illustrated in Figure 3a. In contrast, Zn2 access is restricted by both active site residues and the cephalosporin moiety of FDC. In the EI state, Asn220 blocks the Zn1 water‐entry pathway by forming a hydrogen bond with the ring‐opened β‐lactam carboxylate group of FDC, which coordinates with Zn1 and occupies the spatial gap. Conversely, the EI state creates space near Zn2, allowing a new channel to form, referred to as the Zn2 water channel (Figure 3b). Water molecules in the Zn1 water channel during the ES state do not directly participate in hydrolysis but likely contribute to active site stabilization. By comparison, a water molecule in the EI‐state Zn2 water channel could directly facilitate hydrolysis by donating a proton to the anionic nitrogen in the EI state. This water molecule plays an essential role in the NDM‐1 catalytic mechanism and is referred to as Pre‐W1 in the EI state.

Protonation of the anionic nitrogen by Pre‐W1 in the EI state serves two key functions in the NDM‐1 catalytic process. First, this protonation disrupts the coordination between the anionic nitrogen and Zn2, a characteristic feature of the EI state. Protonation of the FDC ring‐opened β‐lactam carboxyl group by Asp124, together with this nitrogen protonation, results in the dissociation of FDC from both Zn1 and Zn2 in the EP state (Figure 1e). Second, this same protonation event converts Pre‐W1 into a hydroxide anion, which coordinates both Zn1 and Zn2 in the EP state, serving as W1 in the subsequent NDM‐1 catalytic cycle.

Together, these results demonstrate that Zn coordination, FDC conformational changes, and key active site residues, particularly Asp124 and Asn220, jointly regulate water dynamics at the catalytic site. Loop regions, including L10, may also contribute to this process. The observed shift in water distribution may facilitate bulk water delivery during FDC hydrolysis by NDM‐1 and could represent a general mechanism underlying MBL catalysis more broadly. Next, we examine how conformational changes in loops L3 and L10 influence water entry.

### 2.6 Water distribution and conformational dynamics of L3 and L10

Hydrophobic L3 residues are more hydrated in the EI state than in the ES state, as shown by an increased average number of water molecules within 3 Å of key L3 residues (Table 2; see also Table S4 for an extended list). This observation is consistent with the formation of the Zn2 water channel, which involves L3, in the EI state. Conversely, hydration of L10 in the ES state is residue‐dependent, with most residues showing higher hydration and others exhibiting the opposite trend. This indicates a heterogeneous hydration pattern in L10, which may be associated with the presence of the Zn1 water channel.

**TABLE 2:** Average number of water molecules within 3 Å of key residues in the ES and EI states of the NDM‐1–FDC complex, and the differences between these states (∆Water_EI‐ES_).

Residues	ES	EI	∆Water_EI‐ES_
Leu65 (L3)^a^	3.7	4.0	0.3
Met67 (L3)	3.0	4.3	1.3
Phe70 (L3)	6.9	8.7	1.8
Val73 (L3)	2.5	3.8	1.3
Trp93	2.1	3.8	1.7
His120	0.6	0.2	−0.4
His122	4.4	3.0	−1.4
Gln123	6.7	6.2	−0.5
Asp124	1.1	3.8	2.7
His189	3.1	1.6	−1.5
Cys208 (L10)	2.0	0.9	−1.1
Lys211 (L10)	3.8	5.2	1.4
Asp212 (L10)	3.9	5.6	1.7
Leu218 (L10)	5.9	3.4	−2.5
Asn220 (L10)	5.1	4.2	−0.9
His250	3.9	3.9	0.0
FDC	21.0	22.5	1.5
Zn1	1.2	0.1	−1.1
Zn2	0.1	1.0	0.9

^a^ Labels in parentheses represent key residues for loops L3 and L10 used in the analysis of center‐of‐mass (COM) distance distributions.

**FIGURE 5:**
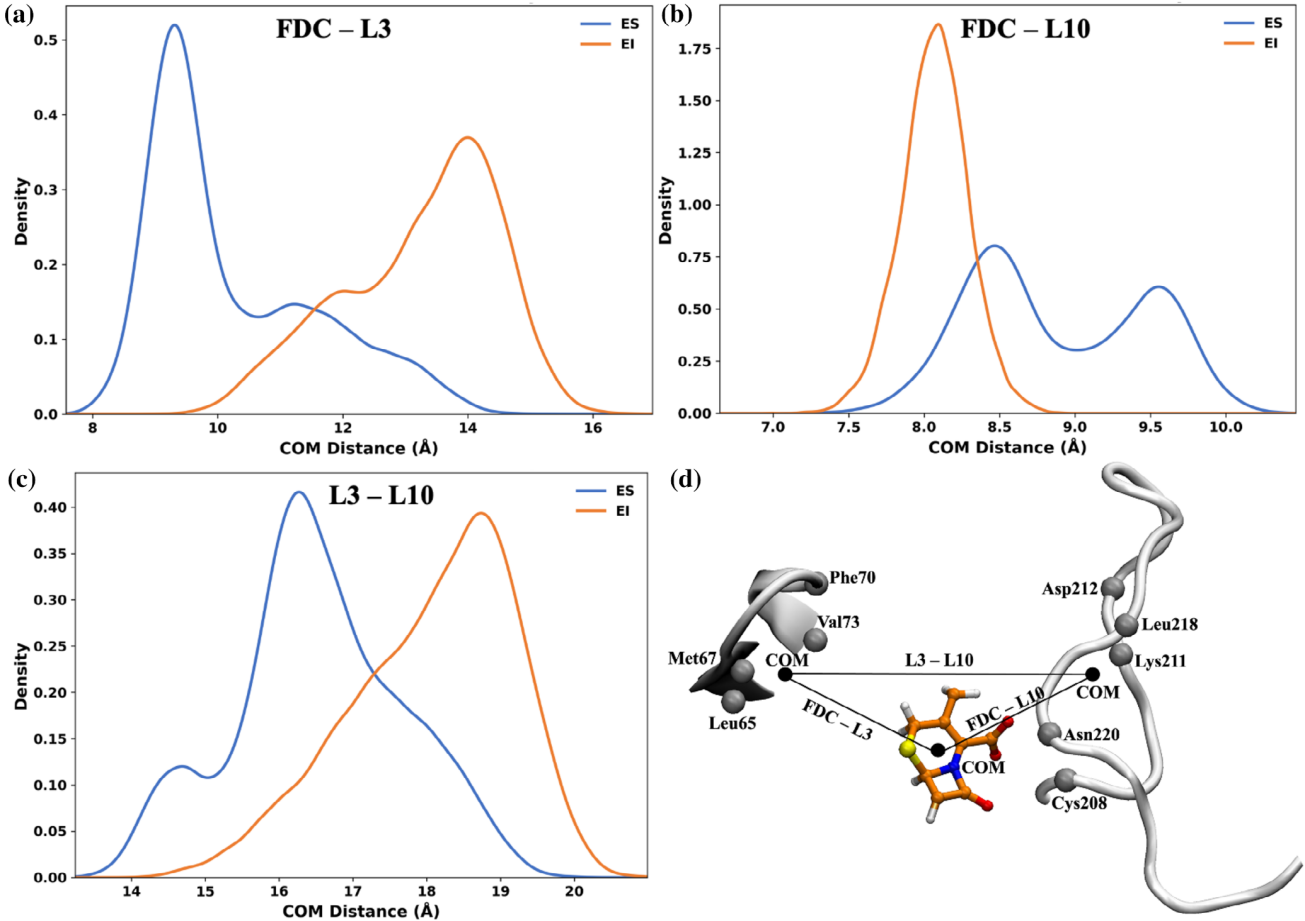
Distributions of center of mass (COM) distances between the cephalosporin moiety of FDC and the L3 and L10 loops. (a–c) COM distances between (a) the cephalosporin moiety of FDC and L3, (b) the cephalosporin moiety of FDC and L10, and (c) L3 and L10. (d) Structural representation showing the cephalosporin moiety of FDC, the Cα atoms of key residues in L3 and L10, and their respective COMs.

To further probe how FDC orientation influences L3 and L10, we measured the relative motions between its cephalosporin moiety and the loop regions. We plotted the distance distribution between the center of mass (COM) of the cephalosporin moiety and the COMs of L3 and L10 (Figure 5a,b). For these calculations, only the backbone Cα atoms of key residues in L3 and L10, defined as residues within 6 Å of either the Zn ions or the cephalosporin moiety of FDC, were included. The selected residues are listed in Table 2. The resulting distance represents the average spatial relationship between FDC and the loops. The distance between FDC and L3 increases by approximately 4.5 Å in the EI state, due to water accumulation between L3 and Zn2, leading to increased separation. In contrast, the FDC–L10 distance decreases as L10 shifts toward FDC in the EI state. The bimodal peaks of the FDC‐L10 distance distribution in the ES state (Figure 5b) correlate with those of the His250–Zn2–FDC angle distribution (Figure 4) and with changes in the number of water interactions with Zn1, which are linked to the Zn1 water channel located near L10 (Figure 2f). This contraction is accompanied by a reduction in hydration between L10 and FDC. Since Zn1 lies in the L10 direction, these hydration patterns directly influence Zn1's solvation. Figure 5c shows that the COM distance between L3 and L10 increases from approximately 16 Å in the ES state to approximately 19 Å in the EI state, reflecting the expansion of the FDC–L3 distance. This shift accommodates the extended Zn2 water channel along L3. These hydration‐driven shifts alter water distribution, leading to a more extensive network near Zn2 than Zn1.

We applied perturbation relative entropy (PRE) analysis to assess changes in Cα pairwise distance distributions between the ES and EI states. PRE revealed significant shifts in distance distributions associated with the EI state, suggesting potential allosteric effects. To identify the structural basis of these changes, we applied relative entropy‐based dynamical allosteric network (REDAN) analysis (Zhou & Tao, 2019) to Cα distance pairs from the active site, L3, and L10. The top three residue pairs with the greatest distribution shifts are Asn220‐Tyr229 and Ile210‐Asn220 (both in L10), and Cys208‐Asn220 (Figure S7). All three pairs involve L10 and the key regulatory residue Asn220, underscoring its role in modulating the water network. Because Cys208 coordinates with Zn2, the Cys208‐Asn220 pair suggests a structural link between L10 and Zn2. Ile210 forms a hydrogen bond with the catechol hydroxyl group of FDC in the EI state, indicating coupling between L10 and FDC. Tyr229 forms a transient hydrogen bond with a catechol hydroxyl group of FDC in the ES state, but not in the EI state. The top 10 PRE values are listed in Table S5.

These results suggest that the formation of the anionic intermediate directly influences water distribution and conformational shifts in L3 and L10. As a result, water preferentially diffuses toward Zn2 over Zn1, positioning solvent near the anionic nitrogen and potentially facilitating its protonation. To further characterize these changes, we applied MSM analysis, which identified discrete states and transitions that capture loop rearrangements during the ES‐to‐EI conversion of NDM‐1.

### 2.7 Kinetic landscape of NDM‐1 states revealed by Markov state model

The conformational dynamics of NDM‐1 were analyzed using an MSM. Simulation frames were clustered into microstates and grouped into metastable macrostates to capture the system's kinetic behavior. For each state of NDM‐1 (NDM‐1, ES, EI, and EP), we used the implied timescales to determine the optimal lag time and an appropriate number of metastable states (Figures S8 and S9; Table S6). Table S7 lists the microstates and their corresponding frames for each macrostate.

The metastable distributions differed markedly: the ES ensemble consisted of five macrostates, whereas the EI ensemble consisted of four (Figure S10A,B). These differences reflect rearrangements in the active site and in loops L3 and L10. Representative structures from each state highlight distinct L3 and L10 conformations across states, with macrostate population gradients illustrated in Figure S11 using a color scale from blue (high) to white (low) population density. These conformations indicate that L3 shifts away from FDC, while L10 moves closer, consistent with rearrangements facilitating water entry in the EI state. MSM analysis reveals that these conformational changes enable water access to the active site near Zn2. Transition probability matrices indicate that NDM‐1 remains globally rigid. However, the EI state shows higher transition rates than the ES state, consistent with stabilization by additional hydrogen bonds and coordination of the anionic nitrogen (Figure S10C,D). The stationary distribution identified metastable state S5 as the most populated in the ES state (Figure S10C, structure in yellow) and metastable state S3 as the most populated in the EI state (Figure S10D, structure in green).

**FIGURE 6:**
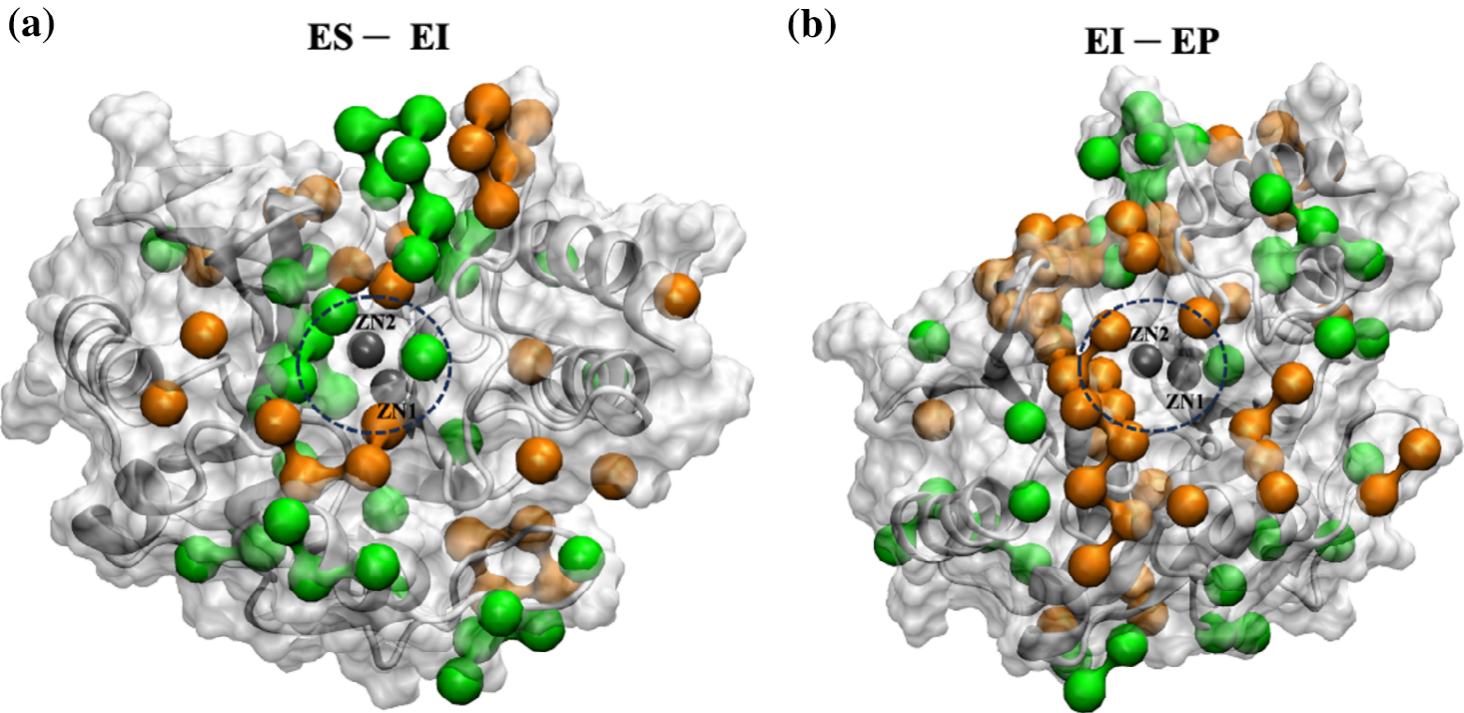
Changes in water distribution during the transitions. (a) From ES to EI and (b) from EI to EP. Green denotes regions of increased water density, whereas orange denotes regions of decreased water density.

To further investigate hydration changes, we performed a grid‐based water analysis comparing the ES state and the EI state (see Supporting Information Materials and Methods for details). Water occupancy differences (∆Water _EI‐ES_) revealed minimal changes across the protein surface, with significant redistribution near the active site (Figure 6). Water distributions were mapped and compared between the ES–EI and EI–EP states, with spatial occupancy patterns provided in Figure S12. Compared to the ES state, the EI state shows increased water density around Zn2, reflecting enhanced hydration, whereas water occupancy around Zn1 is reduced (Figure 2d). In contrast to the EI–ES comparison, the EI‐to‐EP comparison shows negative grid cells around Zn2, consistent with water depletion at this site in the EP state. These findings indicate that the EP state adopts a distinct hydration pattern.

These hydration changes correlate with conformational rearrangements in L3 and L10. Together, these features support a mechanism in which water diffuses toward Zn2 in the EI state, enabling protonation at N2^−^ and thus completing the catalytic cycle of the NDM‐1–FDC complex. After hydrolysis, water distribution shifts again, and the hydrolyzed FDC is displaced from the active site in the EP state.

## 3 CONCLUSIONS

The hydrolysis of FDC by NDM‐1 proceeds in two steps: (i) the bridging hydroxide (W1) attacks the β‐lactam carbonyl carbon, cleaving the carbon–nitrogen (C1–N2) bond and forming an anionic intermediate (N2^−^), accompanied by proton transfer to Asp124; and (ii) a bulk water protonates N2^−^ and regenerates the catalytic hydroxide bridging the zinc ions. The crystallographic Zn2‐coordinated water (W2) is not directly involved in catalysis and may instead hinder EI formation. Catalysis is governed by dynamic water access, with a shift in water channel preference from Zn1 in the ES state and Zn2 in the EI state.

Following nucleophilic attack, the intermediate contains an anionic nitrogen and a ring‐opened β‐lactam carboxylate group that coordinate strongly with the zinc ions. This ring‐opened intermediate is associated with an increased Zn1–Zn2 distance and expansion of the active site. These changes promote dehydration at Zn1 and formation of a water channel near Zn2, facilitating proton transfer and product release. Analyses of L3 and L10 dynamics, Markov state models of the ES and EI states, and grid‐based water population mapping consistently support an alternating water channel mechanism. These findings provide molecular‐level insight into how zinc coordination and loop dynamics regulate water access in the NDM‐1–FDC complex and may inform the rational design of inhibitors targeting water‐mediated catalysis.

## 4 MATERIALS AND METHODS

### 4.1 Molecular docking

Since no crystal structure of the NDM‐1–FDC complex is available, molecular docking was used to generate the initial binding conformation. NDM‐1 (PDB ID: 5ZGZ) was prepared by removing alternate B conformations, and protonation states were assigned using the H++ server (Gordon et al., 2005). Docking was performed with AutoDock Vina (Nguyen et al., 2020), incorporating a catalytic water (W2) and bridging hydroxide (W1) in the active site. The final NDM‐1–FDC conformation was selected based on key interactions observed in the NDM‐1–meropenem complex (Tripathi & Nair, 2015), including the positioning of the β‐lactam ring near the zinc ions and proper orientation of W1 to support nucleophilic attack that initiates catalysis. This conformation was validated for mechanistic consistency and used as the starting structure for subsequent simulations.

### 4.2 Molecular mechanics (MM) modeling

The NDM‐1–FDC complex was subjected to MM energy minimization in the presence or absence of a water molecule (W2) coordinated to Zn2, as in prior studies (Yang et al., 2014). The complex was solvated in a cubic water box (75 × 75 × 75 Å^3^) using the TIP3P water model. The CHARMM36 force field (Huang & Mackerell, 2013) was used for NDM‐1, and CGenFF (Vanommeslaeghe et al., 2010) for FDC. Atomic partial charges for cefiderocol were derived using the restrained electrostatic potential (RESP) method at the HF/6‐31G(d, p) level of theory, as implemented in Antechamber (Bayly et al., 1993). To neutralize the system, sodium (Na^+^) and chloride (Cl^−^) ions were added. Periodic boundary conditions were applied. All bonds involving hydrogen atoms were constrained using the SHAKE algorithm (Ryckaert & Ciccotti, 1977). Nonbonded interactions were treated with a 12 Å cutoff, and long‐range electrostatic interactions were calculated using the particle mesh Ewald (PME) method (Darden et al., 1993). Energy minimization was performed for 5000 steps using the adopted basis Newton–Raphson (ABNR) algorithm, followed by 1 ns equilibration under isothermal‐isobaric (NPT) conditions. The equations of motion were integrated using a leapfrog algorithm (Van Gunsteren & Berendsen, 1988) with a 2 femtosecond (fs) time step. All minimization and equilibration were carried out using CHARMM (Brooks et al., 2009).

### 4.3 Modeling EI and EP states

The equilibrated NDM‐1–FDC structures with and without W2 were further optimized using a QM/MM approach. Because the complex containing W2 was catalytically unfavorable, the NDM‐1–FDC complex without W2 was considered the ES state. Based on this ES structure, the EI and EP states were subsequently modeled and optimized. Residues within 6 Å of the active‐site zinc ions (Leu65, Met67, Phe70, Val73, Trp93, His120, His122, Asp124, His189, Cys208, Lys211, Asn220, Met248, His250) were included in the QM region. To capture the effects of the second‐shell coordination sphere (SCS) on substrate binding and transition state stabilization (Chaturvedi et al., 2023), a large QM region comprising 264 atoms, including FDC and two active‐site zinc ions (Zn1 and Zn2), was employed. The mobile MM region, comprising all residues within 10 Å of the QM atoms, was allowed to move during the QM/MM calculations, whereas the remainder of the MM region was fixed. The QM region was treated using the self‐consistent‐charge density‐functional tight‐binding (SCC‐DFTB) method with the 3OB parameter set (Gaus et al., 2014), while the MM region was modeled with the CHARMM36 force field (Huang & Mackerell, 2013). Hydrogen link atoms were placed between Cα−Cβ bonds to define the QM/MM boundary.

To model the EI complex, optimization was performed with restraints on the following distances: OW1–C18, C18–N2^−^, HW1–COO^−^(Asp124), and N2^−^–Zn2. For the EP complex, restraints were applied to: H_bulk–N2^−^, HW1–OW1, OH_bulk–Zn1, and OH_bulk–Zn2 distances, followed by optimization (see Figure S13 for details). The QM subsystem was assigned a net charge of +1, consistent with the electronic states of the selected residues. Self‐consistent charge convergence was achieved with a tolerance of 1 × 10^−8^, and an electronic temperature of 300 K was applied. The third‐order expansion of DFTB3, with a hydrogen‐bond correction, was applied to improve charge fluctuations and hydrogen‐bond interactions. Anderson charge mixing was applied to stabilize the self‐consistent field (SCF) iterations and accelerate convergence.

QM/MM geometry optimizations were performed for 5000 steps using the ABNR method for all NDM‐1–FDC states. Convergence was defined by total energy changes of less than 1 × 10^−6^ kcal/mol between successive steps. The stability of the optimized structures was further validated by monitoring key active site distances and substrate positioning. Importantly, the optimized ES, EI, and EP structures preserved catalytically relevant geometries, including proper zinc coordination, β‐lactam ring positioning, and W1, confirming the reliability of the QM/MM models. All QM/MM simulations for the ES, EI, and EP states were performed using the SCC‐DFTB/CHARMM method. Previous studies have shown that this approach reliably reproduces energy barriers consistent with experimental data for hydrolysis reactions (W. Jitonnom, Wanjai, & Jitonnom, 2025; W. Jitonnom, Wanjai, Friedman, et al., 2025; Meelua et al., 2023; Meelua, Wanjai, et al., 2022).

### 4.4 Molecular dynamics (MD) simulation

To probe water network dynamics, extensive MD simulations were conducted for the NDM‐1, ES, EI, and EP states using structures derived from QM/MM optimizations. All production runs were performed using OpenMM (Eastman et al., 2024). To preserve Zn‐residue coordination, harmonic restraints were applied between zinc ions and coordinating residues, as the CGenFF force field does not fully preserve these interactions. Simulations were conducted at 300 K and 1 bar using Langevin dynamics coupled with a Monte Carlo barostat (Izaguirre et al., 2010; Kong et al., 2003). Each production simulation was 1 μs in length, with a 2 fs time step and trajectory frames saved every 100 ps, yielding 10,000 frames per simulation. For each state, three independent simulations were performed and combined for analysis.

The representative structures used to illustrate the water channels (Figure 3) and water distributions related to catalysis (Figure 6) were selected from the simulations as follows. For each state (ES, EI, and EP), all frames were first superimposed onto a reference structure using least‐squares fitting based on RMSD. An average structure was then calculated from the superimposed frames. Because this average structure does not represent an actual sampled conformation, it was used only as a reference to identify a representative structure from the original trajectory. The frame with the smallest RMSD relative to the average structure was selected for each state and used for visualization.

### 4.5 Markov state models

Markov state models (MSMs) were constructed to characterize metastable states of the NDM‐1, ES, EI, and EP forms of the NDM‐1–FDC complex using PyEMMA 2.5.12 (Prinz et al., 2011; Scherer et al., 2015). For each state, 150,000 frames from three independent simulations were analyzed. Given the overall rigidity of the NDM‐1 structure, except for the more dynamic active site residues within 6 Å of the zinc ions, careful feature selection was essential. Pairwise Cα–Cα distances among residues in the active site, as well as the L3 and L10 loops, were selected, as these regions exhibit greater flexibility and play key roles in substrate binding.

The selected features were projected into time‐lagged independent component analysis (tICA) space to capture the slowest collective motions while preserving kinetic information (Naritomi & Fuchigami, 2011). The first four independent components (ICs) were retained for *k*‐means clustering to define microstates. To confirm Markovian behavior, implied timescales were plotted as a function of lag time, and the point of convergence was selected as the optimal lag time. The Chapman–Kolmogorov (CK) test was then used to validate the model (Breuer & Petruccione, 1995). Using this optimal lag time (τ), MSMs were constructed. The microstates were grouped into macrostates via Perron Cluster Cluster Analysis (PCCA++) (Röblitz & Weber, 2013). The number of macrostates was determined by inspecting gaps in the dominant implied timescales. Trajectory visualizations were generated using Visual Molecular Dynamics (VMD) (Ricker, 1954) and PyMOL (L DeLano, 2002).

## AUTHOR CONTRIBUTIONS

**Palanisamy Kandhan:** Methodology; software; data curation; investigation; validation; formal analysis; visualization; writing – original draft; writing – review and editing; conceptualization. **Peng Tao:** Conceptualization; methodology; software; investigation; supervision; funding acquisition; project administration; resources; writing – review and editing; writing – original draft. **Chuanye Xiong:** Methodology; software; data curation. **Timothy Palzkill:** Resources; writing – review and editing.

## CONFLICT OF INTEREST STATEMENT

The authors declare no conflicts of interest.

## Supporting information


**Figure S1.** Structural overview of New Delhi metallo‐β‐lactamase 1 (NDM‐1), its active site environment, and the chemical structure of FDC in the ES and EI states.
**Figure S2.** Time evolution of the backbone RMSD for simulations of the NDM‐1–FDC complex in different catalytic states.
**Figure S3.** Side‐chain RMSF of each residue in the NDM‐1–FDC complex in distinct catalytic states.
**Figure S4.** Time evolution of hydrogen bonds between FDC and NDM‐1, along with hydrogen bond occupancy (%) for the ES and EI states.
**Figure S5.** Hydrogen‐bond interactions between FDC and NDM‐1 residues.
**Figure S6.** Number of water molecules in the network near the zinc ions in the ES and EI states based on the simulations.
**Figure S7.** Top three perturbation relative entropy (PRE) values for selected Cα pairs from the active site, L3, and L10.
**Figure S8.** Implied timescales plotted as a function of lag time for the NDM‐1–FDC complex in different catalytic states.
**Figure S9.** Implied timescales plotted versus implied timescale index for the NDM‐1–FDC complex in different catalytic states.
**Figure S10.** Markov state models (MSMs) of the ES and EI states of the NDM‐1–FDC complex.
**Figure S11.** Superimposed representative NDM‐1–FDC structures of macrostates identified in the MSMs.
**Figure S12.** Grid‐based water analysis of the NDM‐1–FDC complex in the ES and EI states.
**Figure S13.** Illustration of the preparation of the NDM‐1–FDC complex in the EI and EP states starting from the ES state.
**Table S1.** Comparison of key distances (Å) in QM/MM–optimized NDM‐1–FDC structure and MM MD‐simulated NDM‐1–meropenem.
**Table S2.** Average backbone RMSD for the NDM‐1–FDC complex in distinct catalytic states.
**Table S3.** Average number of hydrogen bonds and their distances between NDM‐1 residues and FDC in the ES and EI states.
**Table S4.** Average number of water molecules within 3 Å of extended list of residues in the ES and EI states of the NDM‐1–FDC complex.
**Table S5.** Top 10 residue pairs with the highest perturbation relative entropy (PRE) values between the ES and EI states of NDM‐1–FDC.
**Table S6.** Parameters used to construct the Markov state model (MSM) for all NDM‐1 states.
**Table S7.** Number of macrostates in the MSM and their corresponding populations, expressed as the number of microstates and total frames, for all NDM‐1 catalytic states.

## Data Availability

The data for three independent trajectories of the NDM‐1, ES, EI, and EP states are available on Zenodo https://doi.org/10.5281/zenodo.17780303.
