# Supplementary material for: Functional dynamics of water in New Delhi metallo‐β‐lactamase catalysis
Source: Protein Sci. 2026 May 18;35(6):e70633. doi: 10.1002/pro.70633 (PMC13182265; doi:10.1002/pro.70633)
Supplement: Supplementary file 1 — Figure S1. Structural overview of New Delhi metallo‐β‐lactamase 1 (NDM‐1), its active site environment, and the chemical structure of FDC in the ES and EI states. Figure S2. Time evolution of the backbone RMSD for simulations of the NDM‐1–FDC complex in different catalytic states. Figure S3. Side‐chain RMSF of each residue in the NDM‐1–FDC complex in distinct catalytic states. Figure S4. Time evolution of hydrogen bonds between FDC and NDM‐1, along with hydrogen bond occupancy (%) for the ES and EI states. Figure S5. Hydrogen‐bond interactions between FDC and NDM‐1 residues. Figure S6. Number of water molecules in the network near the zinc ions in the ES and EI states based on the simulations. Figure S7. Top three perturbation relative entropy (PRE) values for selected Cα pairs from the active site, L3, and L10. Figure S8. Implied timescales plotted as a function of lag time for the NDM‐1–FDC complex in different catalytic states. Figure S9. Implied timescales plotted versus implied timescale index for the NDM‐1–FDC complex in different catalytic states. Figure S10. Markov state models (MSMs) of the ES and EI states of the NDM‐1–FDC complex. Figure S11. Superimposed representative NDM‐1–FDC structures of macrostates identified in the MSMs. Figure S12. Grid‐based water analysis of the NDM‐1–FDC complex in the ES and EI states. Figure S13. Illustration of the preparation of the NDM‐1–FDC complex in the EI and EP states starting from the ES state. Table S1. Comparison of key distances (Å) in QM/MM–optimized NDM‐1–FDC structure and MM MD‐simulated NDM‐1–meropenem. Table S2. Average backbone RMSD for the NDM‐1–FDC complex in distinct catalytic states. Table S3. Average number of hydrogen bonds and their distances between NDM‐1 residues and FDC in the ES and EI states. Table S4. Average number of water molecules within 3 Å of extended list of residues in the ES and EI states of the NDM‐1–FDC complex. Table S5. Top 10 residue pairs with the highest perturbation relative en [file PRO-35-e70633-s001.pdf]

Supplementary Material for

Functional Dynamics of Water in New Delhi Metallo-β-lactamase Catalysis

Palanisamy Kandhan,[a] Chuanye Xiong,[a] Timothy Palzkill,[b] Peng Tao [a*]

[a]Department of Chemistry, O'Donnell Data Science and Research Computing Institute, Center for Drug Discovery, Design, and Delivery (CD4), Southern Methodist University, Dallas, TX 75275, USA.

[b]Verna and Marrs McLean Department of Biochemistry and Molecular Pharmacology, Baylor College of Medicine, Houston, TX 77030, USA.

* Correspondence to: ptao@smu.edu

Materials and Methods

Grid-based water distribution analysis

A unified protein structure file (PSF) was prepared for the enzyme–substrate (ES), enzyme–intermediate (EI), and enzyme–product (EP) states by removing ions and cefiderocol (FDC) from each structure to enable consistent comparison of protein hydration. Trajectories from the ES and EI states (a total of 60,000 frames) were aligned to a representative EI structure. This structure was obtained by averaging atomic coordinates over the EI trajectories, followed by calculation of backbone root mean square deviation (RMSD) between each frame and the average structure. The frame with the lowest RMSD relative to the average was selected as representative. The aligned trajectories were subsequently merged for grid-based analysis. A three-dimensional (3D) grid with 5 Å spacing was applied across the simulation box (75 × 75 × 75 $Å^3$), resulting in 15 divisions along each axis and a total of 3,375 grid cells. This resolution was chosen to capture both primary and secondary hydration shells.

For each grid cell, the average water occupancy was calculated, and difference between states ($\Delta Water_{EI-ES}$) were determined. Grid cells with absolute $\Delta Water_{EI-ES}$ values larger than 0.5 were selected to identify significant hydration changes (**Figure S12A**). Positive values indicate higher occupancy in the EI state, while negative values indicate higher occupancy in the ES state. To visualize these regions, pseudo-atoms corresponding to selected grid cells were generated and mapped onto the representative EI and EP structures (**Figure 6A and 6B**). The same protocol was applied to compare the EI and EP states.

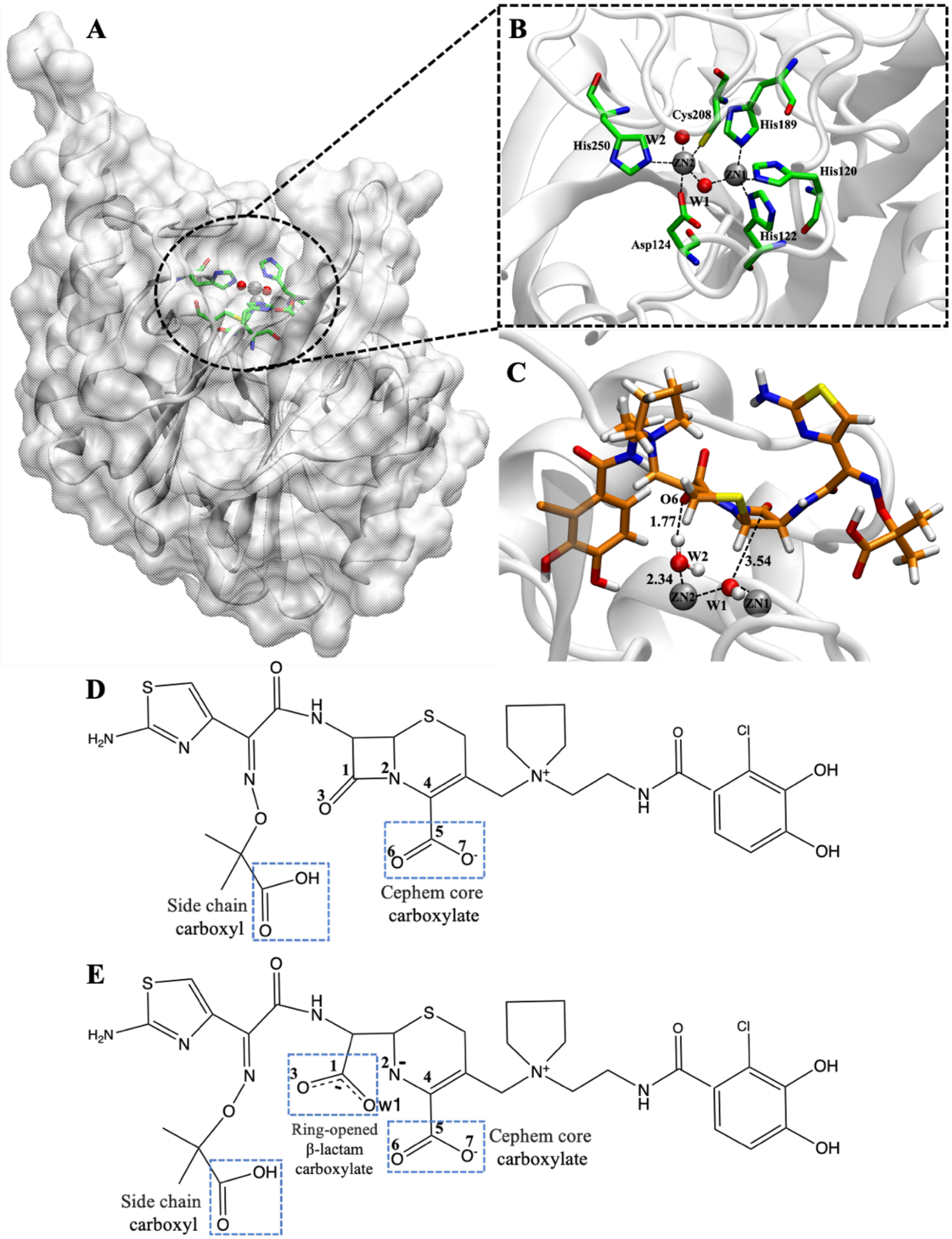

Figure S1. Structural overview of New Delhi metallo-β-lactamase 1 (NDM-1) and cefiderocol (FDC) binding. (A) Crystal structure of NDM-1 (PDB ID: 1ZNB). (B) Active site of NDM-1. (C) Active site of the SCC-DFTB–optimized NDM-1–cefiderocol complex. (D) Chemical structure of FDC in the ES state. (E) Chemical structure of FDC in the EI state. Active site residues are shown as green licorice representations, FDC is shown as an orange licorice representation, and zinc ions are shown as black spheres. Key interactions, including coordination and hydrogen bonds, are indicated by dotted lines with distances measured in Å. Key atoms are labeled as C1, N2, O3, C4, C5, O6, and O7.

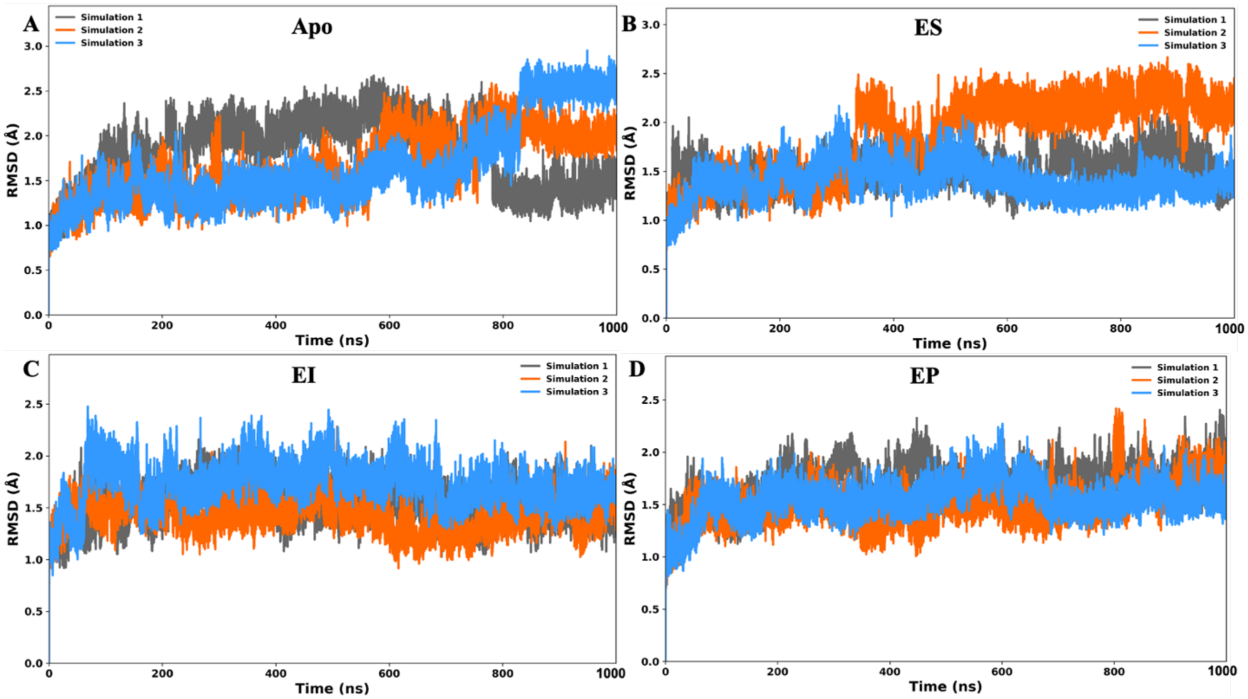

Figure S2. Time evolution of the backbone root mean square deviation (RMSD) for simulations of the NDM-1–FDC complex in different catalytic states: (A) NDM-1, (B) enzyme-substrate (ES) complex, (C) enzyme–intermediate (EI) complex, and (D) enzyme–product (EP) complex.

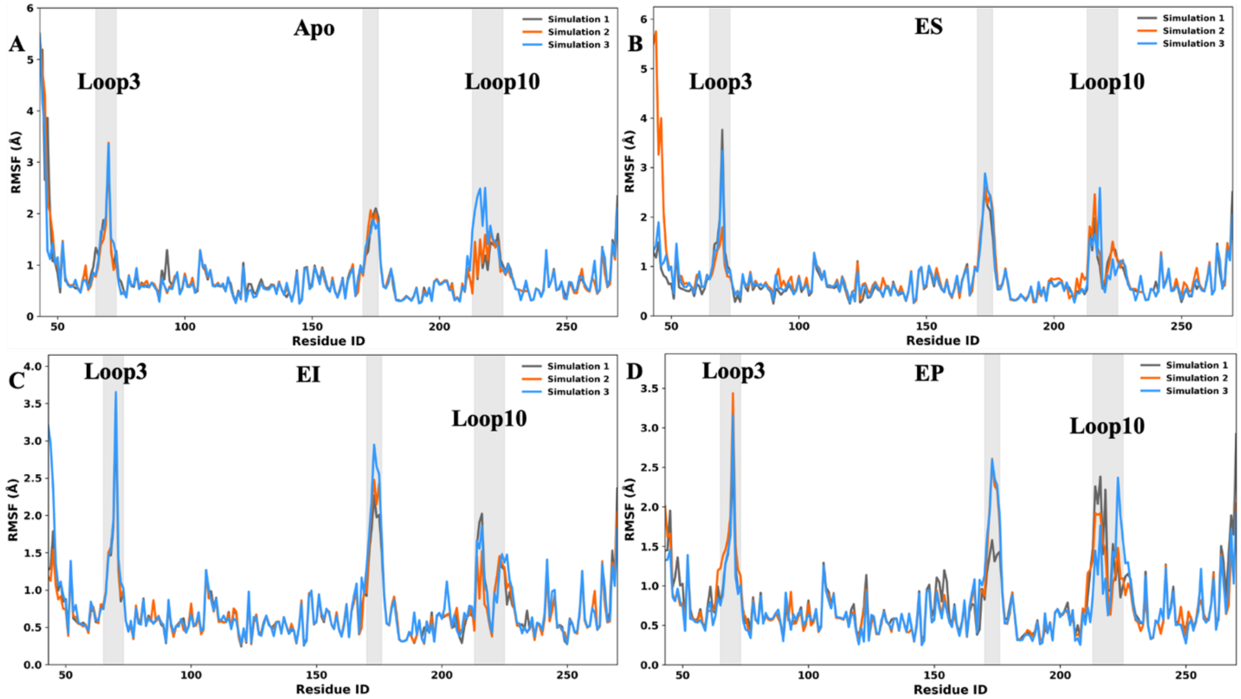

Figure S3. Side-chain root-mean-square fluctuation (RMSF) of each residue in the NDM-1–FDC complex in distinct catalytic states: (A) NDM-1, (B) ES, (C) EI, and (D) EP.

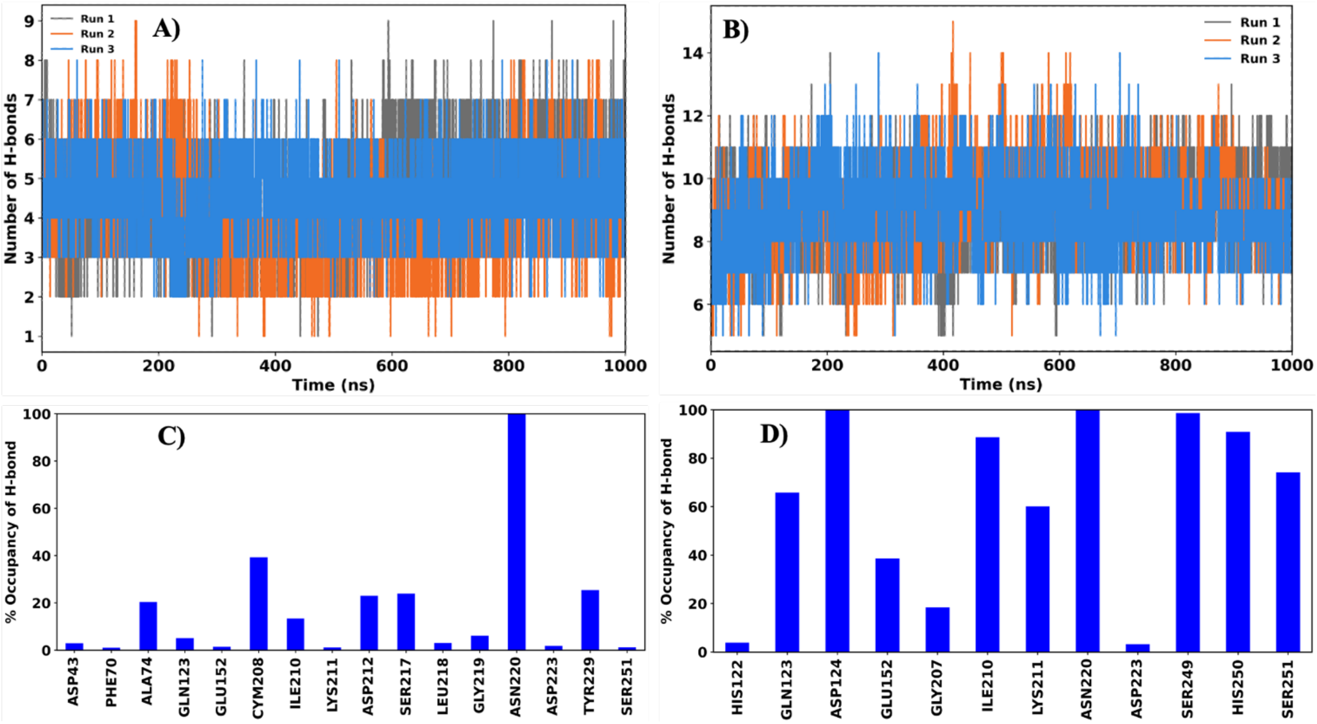

Figure S4. Time evolution of hydrogen bonds between FDC and NDM-1 during the simulations of (A) ES and (B) EI states. Hydrogen bond occupancy (%) for the (C) ES and (D) EI states.

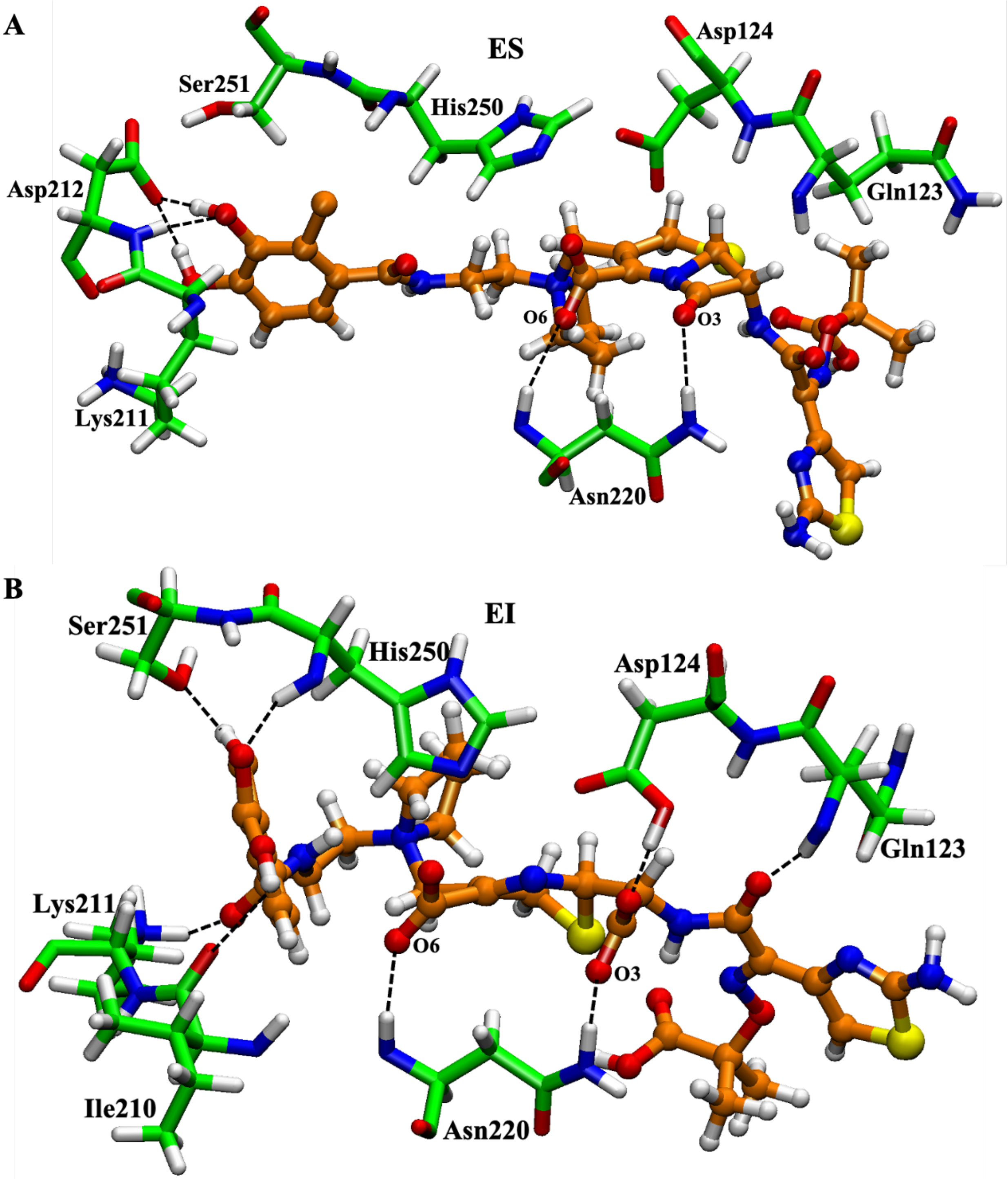

Figure S5. Hydrogen-bond interactions between FDC and NDM-1 residues in (A) the ES state and (B) the EI state. Representative ES and EI structures are shown in Figure 3. Active site (side chains) residues are shown as green licorice representations, and FDC is shown as an orange ball-and-stick representation. Hydrogen bonds between NDM-1 residues and FDC are indicated by dotted lines.

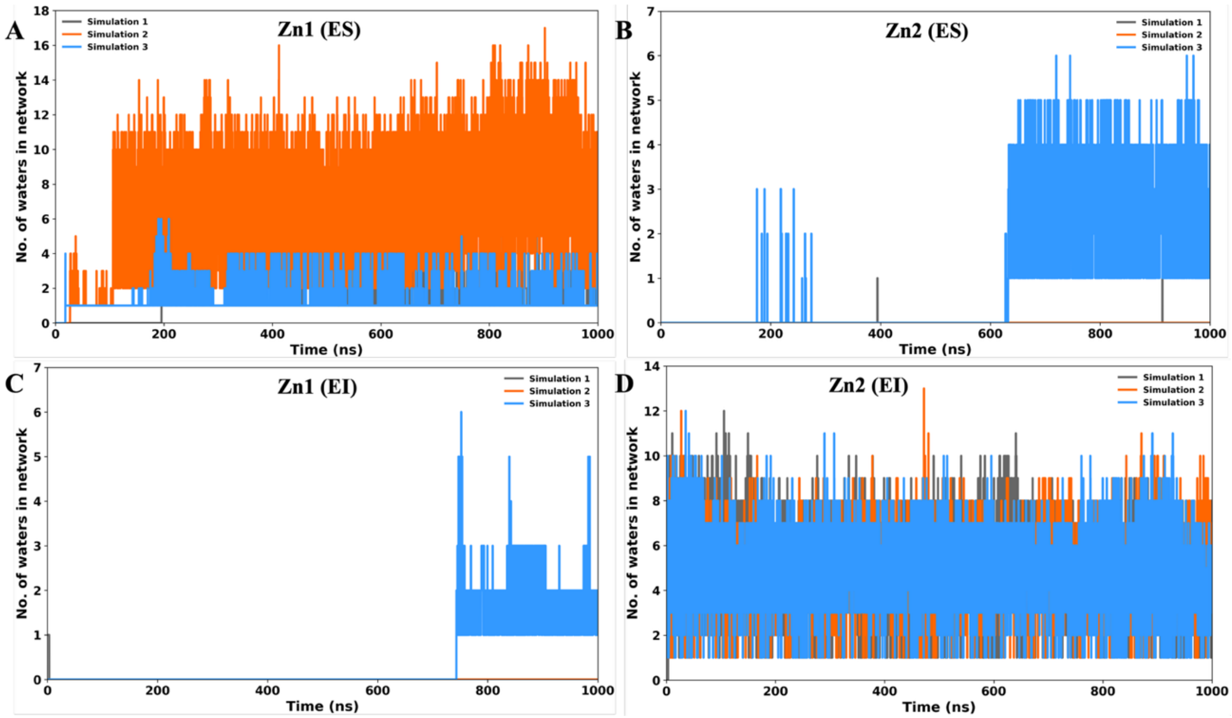

Figure S6. Number of water molecules in the network near zinc ions in the ES and EI states during simulations. (A) Zn1 in the ES state, (B) Zn2 in the ES state, (C) Zn1 in the EI state, and (D) Zn2 in the EI state.

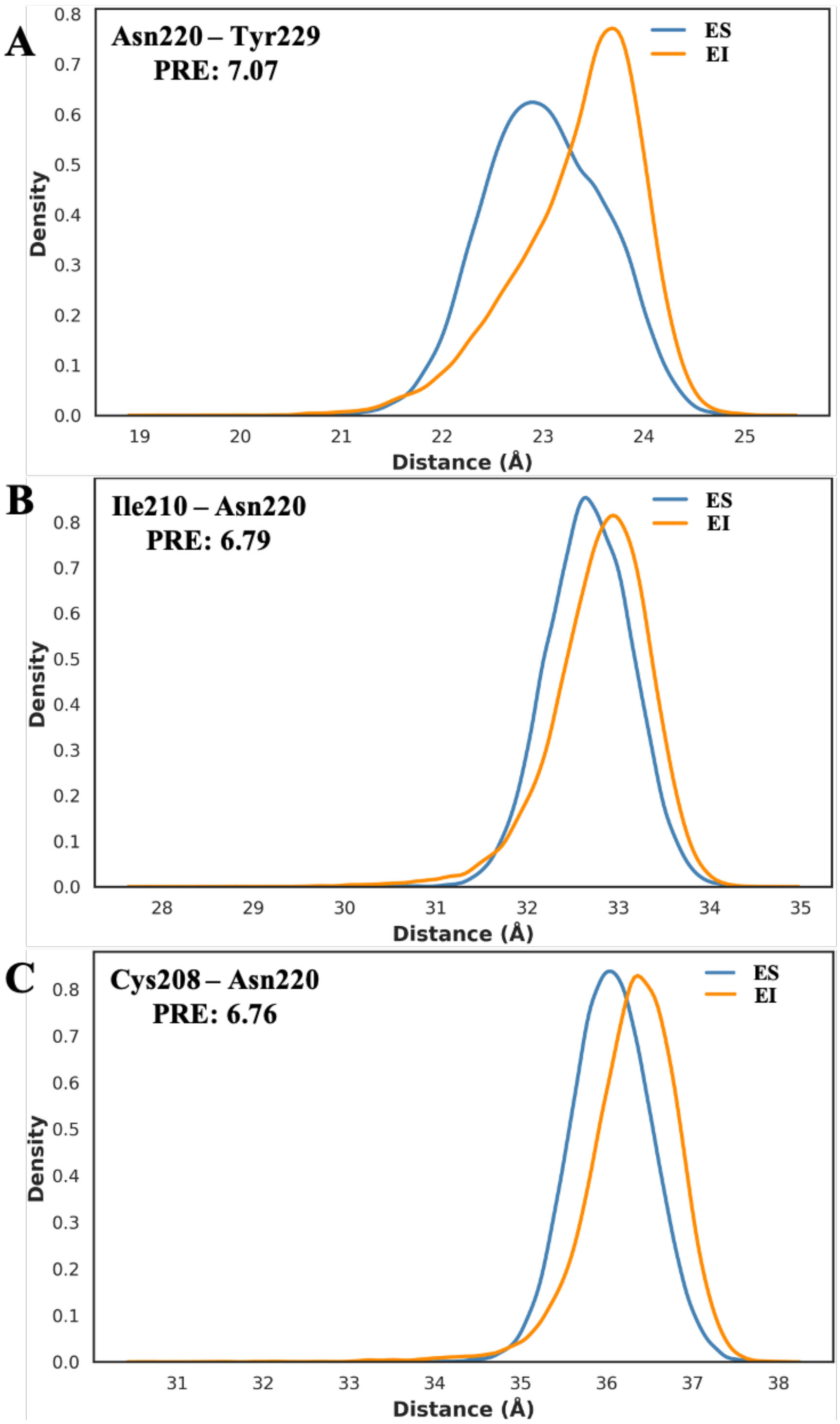

Figure S7. Top three perturbation relative entropy (PRE) values for selected Cα pairs from the active site, L3, and L10: (A) Asn220–Tyr229, (B) Ile210–Asn220, and (C) Cys208–Asn220.

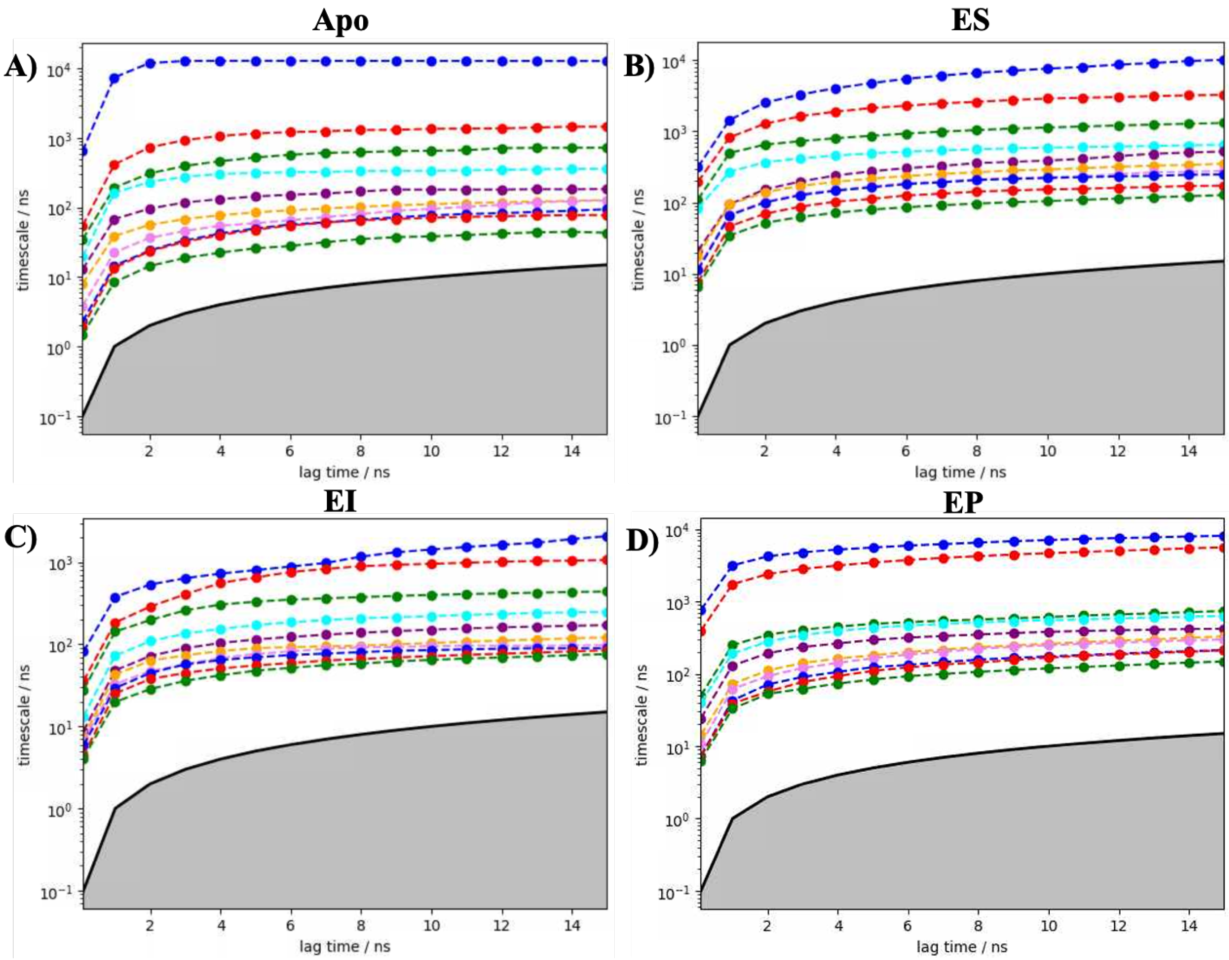

Figure S8. Implied timescales plotted as a function of lag time for the NDM-1–FDC complex in different catalytic states: (A) NDM-1, (B) ES, (C) EI, and (D) EP. The black line and gray-shaded region represent the lag time (τ), used to compute the implied time scale. Implied timescales above the black line correspond to slower processes that are well resolved by the model. In contrast, timescales shorter than the lag time fall within the shaded region and cannot be reliably resolved, as they represent faster processes than the model's temporal resolution.

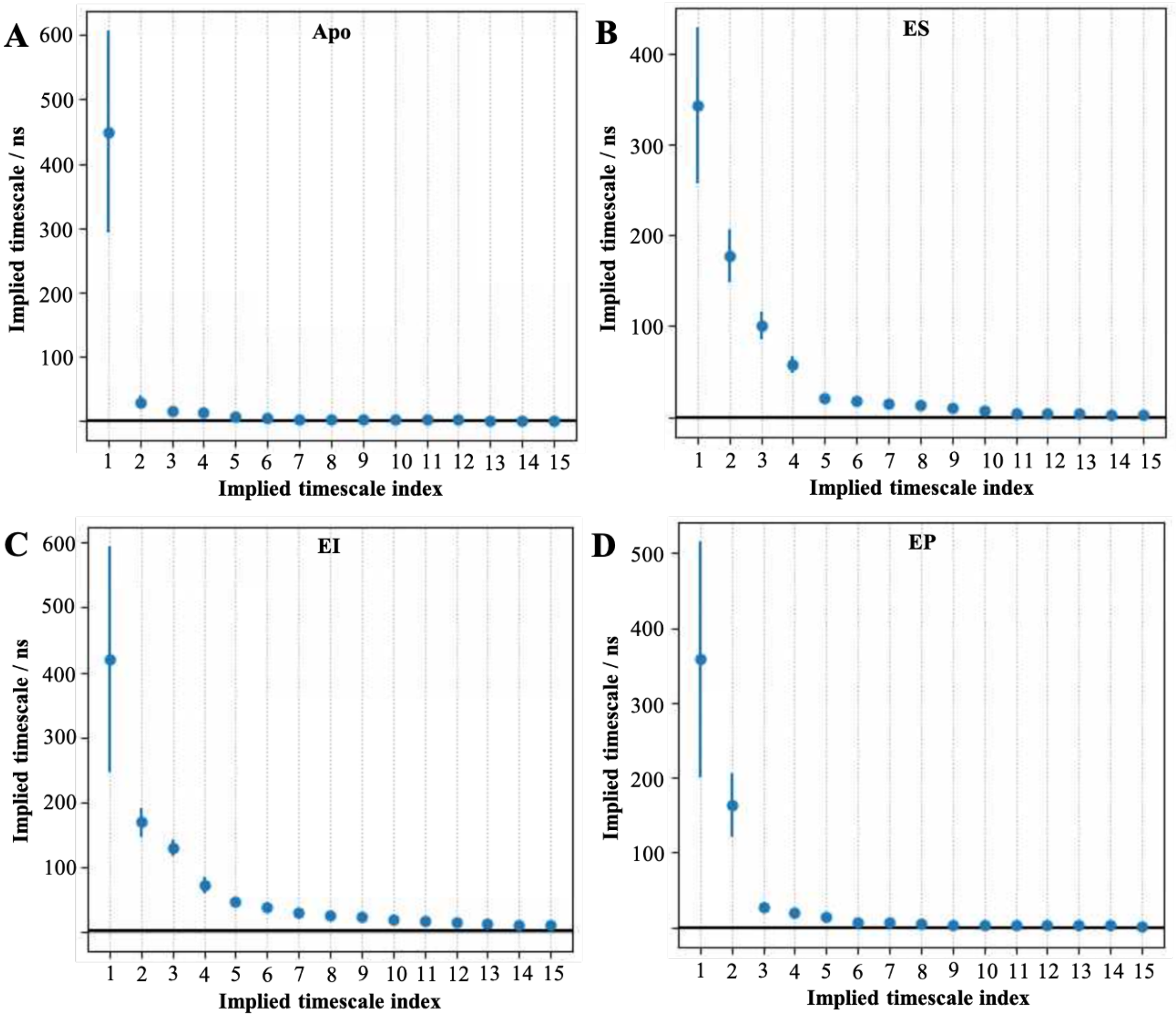

Figure S9. Implied timescales plotted versus implied timescale index for the NDM-1–FDC complex in different catalytic states: (A) NDM-1, (B) ES, (C) EI, and (D) EP. These plots reveal a dominant slow relaxation process, and the separation between the successive timescale suggests an optimal number of metastable states. This choice of the number of metastable states is further supported by the lag-time dependence of the implied time scale shown in Figure S6, where the convergence of the slow modes validates the coarse-graining in the MSM analysis.

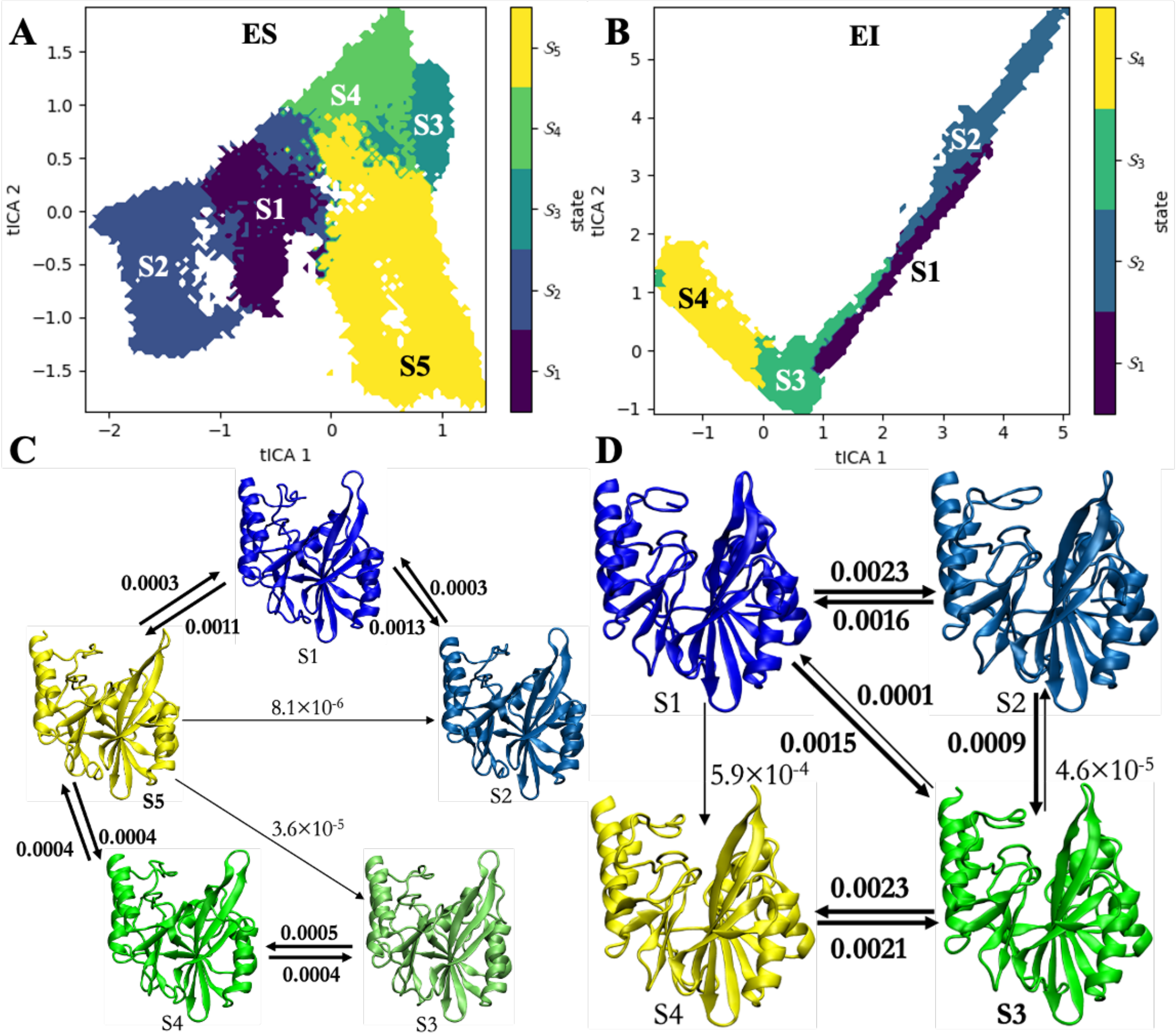

Figure S10. Markov state models (MSMs) of the ES and EI states of the NDM-1–FDC complex. (A, B) Distributions of metastable states for (A) the ES state and (B) the EI state. (C, D) Transition probabilities between metastable states for (C) the ES state and (D) the EI state.

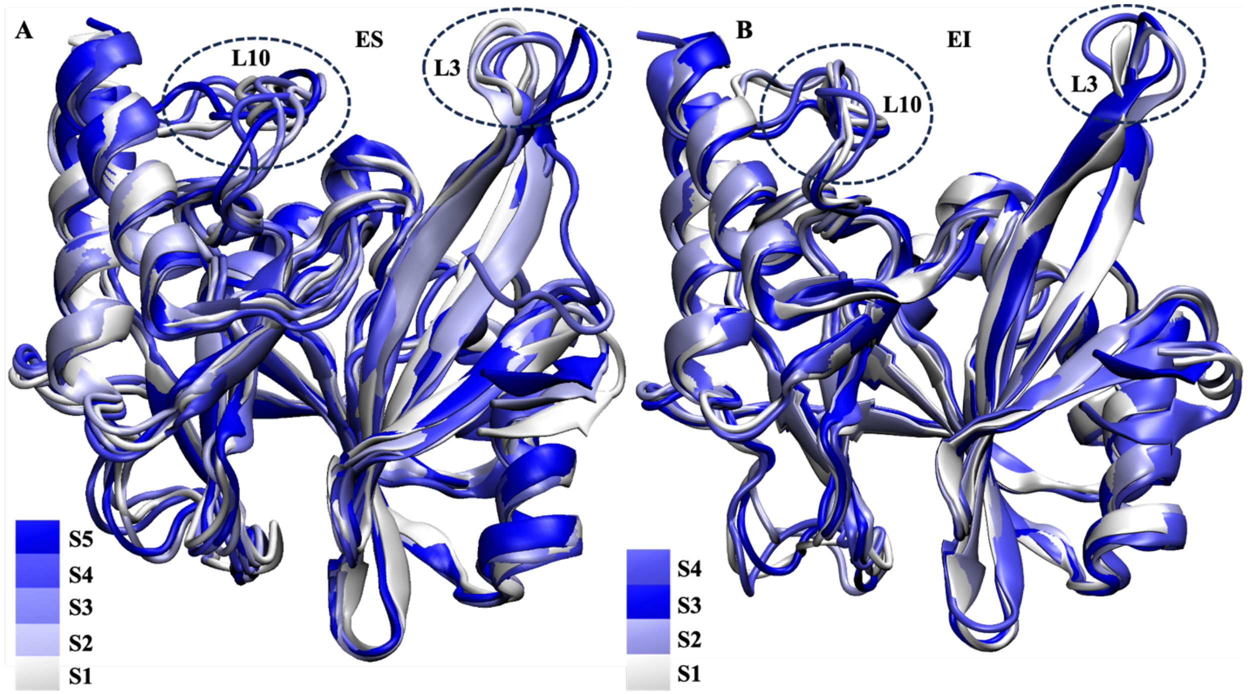

Figure S11. Superimposed representative NDM-1–FDC structures of macrostates identified in the MSMs for the (A) ES and (B) EI states. A blue-to-white color gradient represents decreasing population from high (blue) to low (white).

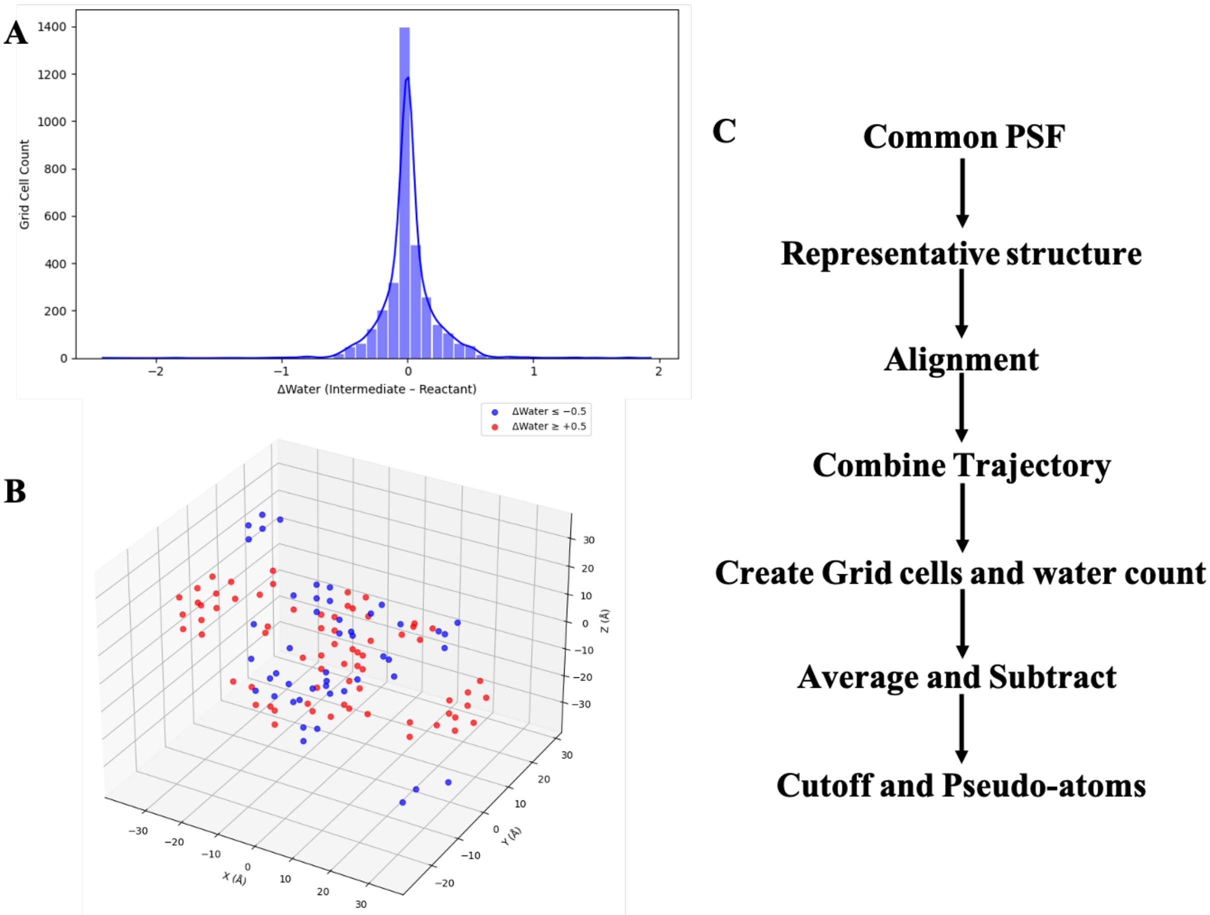

Figure S12. Grid-based water analysis of the NDM-1–FDC complex in the ES and EI states. (A) Water distribution plot showing differences between the two states. (B) Selected grids on the 3D simulation box based on cutoff values. (C) Schematic representation of the analysis method.

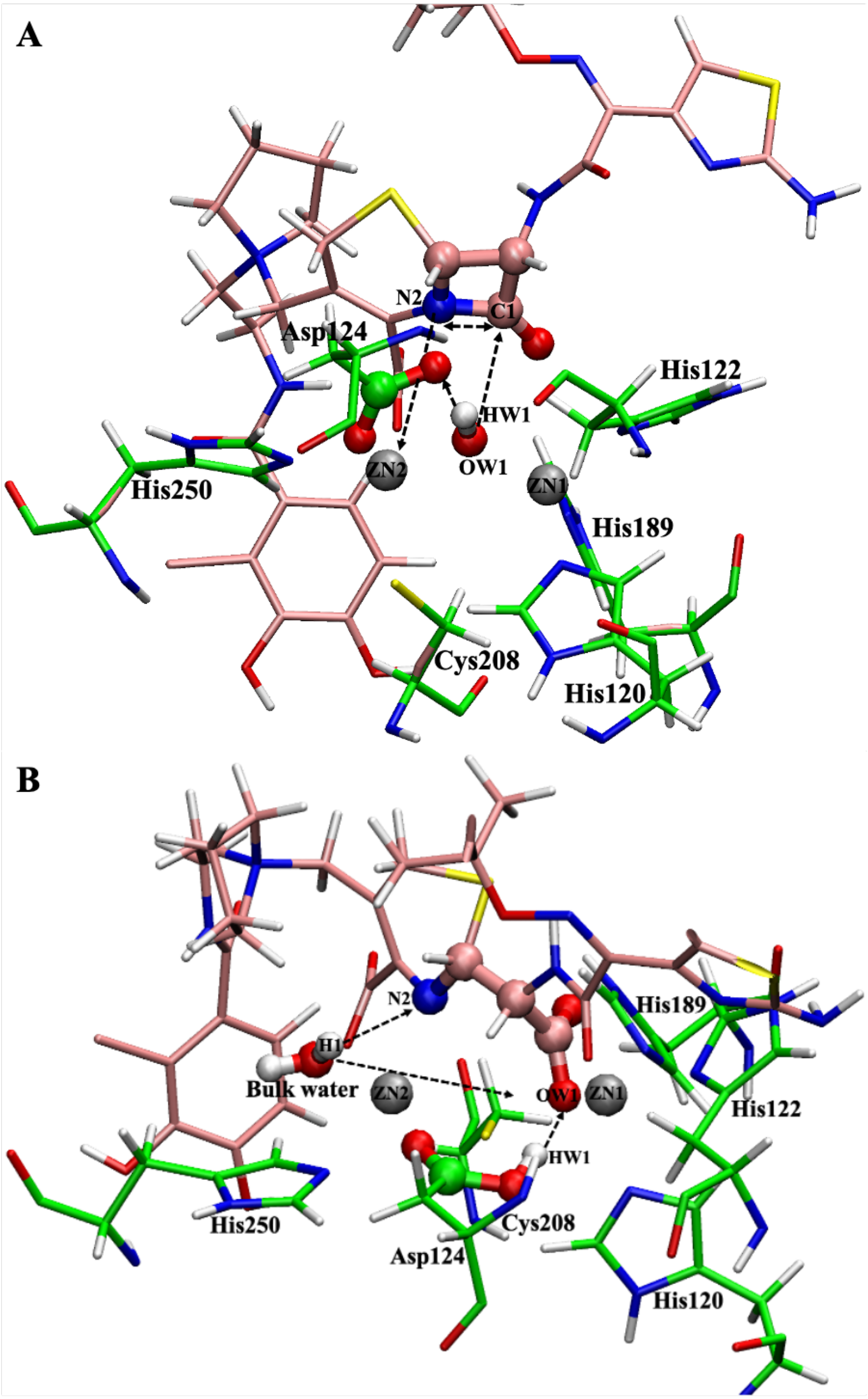

Figure S13. Illustration of the preparation of the NDM-1–FDC complex in the EI and EP states starting from the ES state. Restraint-guided optimization was carried out in two stages: (A) ES to EI and (B) EI to EP. Active site residues are shown as green licorice representations, the cephalosporin core of FDC is shown as orange licorice representation, and zinc ions are shown as black spheres. The distance used to generate the EI and EP models are indicated by dotted lines.

Table S1. Comparison of key distances (Å) in QM/MM–optimized NDM-1–FDC structures and the MM MD–simulated NDM-1–meropenem complex.[1]

Distance	NDM-1–FDC	NDM-1–Meropenem
OW1···C18	3.06	3.10 (± 0.14)
Zn2···O6	2.25	3.69 (± 0.28)
O3···H(Asn220)	1.97	2.01 (± 0.19)
O3···Zn1	3.43	3.35 (± 0.21)
Zn1···Zn2	3.49	3.35 (± 0.09)
O3-N(Asn220)	2.91	3.00 (± 0.40)

Table S2. Average backbone RMSD for the NDM-1–FDC complex in distinct catalytic states.

States of NDM-1–FDC	No. of simulations	Average RMSD (Å)
NDM-1	1	1.81 (0.37) [a]
	2	1.65 (0.36)
	3	1.64 (0.46)
ES	1	1.49 (0.16)
	2	1.85 (0.41)
	3	1.39 (0.17)
EI	1	1.53 (0.17)
	2	1.43 (0.15)
	3	1.72 (0.20)
EP	1	1.68 (0.17)
	2	1.49 (0.19)
	3	1.54 (0.17)

[a] Standard deviations are included in the parentheses.

Table S3. Average number of hydrogen bonds and their distances between NDM-1 residues and FDC in the ES and EI states.

NDM-1 states	Residues	Average No. of hydrogen bonds (Å)	Distances (Å)
ES	Asp212	0.57 (1.11) [a]	2.81 (0.23)
EI	Gln123	0.76 (0.62)	2.98 (0.13)
	Asp124	1.28 (0.44)	2.86 (0.14)
	Ile210	0.89 (0.31)	2.97 (0.21)
	Lys211	0.60 (0.50)	2.74 (0.12)
	Asn220	2.14 (0.40)	2.72 (0.23)
	Ser249	0.99 (0.13)	2.80 (0.14)
	His250	0.90 (0.28)	3.04 (0.12)
	Ser251	0.75 (0.74)	2.95 (0.13)

[a] Standard deviations are included in the parentheses.

Table S4. Average number of water molecules within 3 Å of extended list of residues in the ES and EI states of the NDM-1–FDC complex, and the differences between these states (ΔWater $_{EI-ES}$).

Residues	EI	ES	Δ_{EI-ES}
Leu65 (L3)	4.0	3.7	0.3
Asp66 (L3)	8.9	8.3	0.6
Met67 (L3)	4.3	3.0	1.3
Pro68 (L3)	7.0	6.9	0.1
Gly69 (L3)	4.8	4.8	0.0
Phe70 (L3)	8.7	6.9	1.8
Gly71 (L3)	3.6	3.3	0.3
Ala72 (L3)	4.4	4.3	0.1
Val73 (L3)	3.8	2.5	1.3
Trp93	3.8	2.1	1.7
His120	0.2	0.6	–0.4
His122	3.0	4.4	–1.4
Gln123	6.1	6.7	–0.6
Asp124	3.8	1.1	2.7
His189	1.6	3.1	–1.5
Cys208 (L10)	0.9	2.0	–1.1
Ile210 (L10)	0.1	1.1	–1.0
Lys211 (L10)	5.2	3.8	1.4
Asp212 (L10)	5.6	3.9	1.7
Ser213 (L10)	3.5	4.1	–0.6
Lys214 (L10)	11.1	10.8	0.3
Ala215 (L10)	4.2	5.6	–1.4
Lys216 (L10)	11.9	12.0	–0.1
Ser217 (L10)	5.4	5.5	–0.1
Leu218 (L10)	3.4	5.9	–2.5
Gly219 (L10)	2.3	3.1	–0.8
Asn220 (L10)	4.2	5.1	–0.9
Leu221 (L10)	4.8	5.4	–0.6
Gly222 (L10)	4.4	3.8	0.6
Asp223 (L10)	9.8	9.3	0.5
Ala224 (L10)	4.3	3.3	1.0
Asp225 (L10)	7.4	7.0	0.4
Thr226 (L10)	3.3	4.1	–0.8
Glu227 (L10)	8.7	8.8	–0.1
His228 (L10)	6.0	6.1	–0.1
Tyr229 (L10)	1.4	1.2	0.2
Ala230 (L10)	1.0	1.4	–0.4
His250	3.9	3.9	0.0
FDC	22.5	21.0	1.5
Zn1	0.1	1.2	–1.1
Zn2	1.0	0.1	0.9

Table S5. Top 10 residue pairs with the highest perturbation relative entropy (PRE) values, reflecting changes in Cα pairwise distance distributions between the ES and EI states of the NDM-1–FDC complex.

Rank	Residue pairs	PRE values
1	Asn220:Tyr229	7.07
2	Ile210:Asn220	6.79
3	Cys208:Asn220	6.76
4	Leu221:His250	6.27
5	Gly219:Ile210	5.97
6	His250:His189	5.61
7	Asn220:His228	5.10
8	Trp93:Asn220	5.02
9	His250:Ser217	5.00
10	Leu221:Trp93	4.85

Table S6. Parameters used to construct the Markov state model (MSM) for all NDM-1 states.

NDM-1 states	Microstates	Lag-time (ps)	Macrostates
NDM-1	500	120	3
ES	300	200	5
EI	300	160	4
EP	200	200	4

Table S7. Number of macrostates in the MSM and their corresponding populations, expressed as the number of microstates and total frames, for the NDM-1–FDC complex in the NDM-1, ES, EI, and EP states.

NDM-1 States	Macrostates	No. of Microstates	No. of Frames
NDM-1	S1	82	10343
	S2	68	9255
	S3	350	130402
ES	S1	51	15704
	S2	55	35212
	S3	62	37955
	S4	28	12056
	S5	104	49073
EI	S1	18	3190
	S2	24	4996
	S3	132	95507
	S4	117	45605
EP	S1	25	14652
	S2	37	26332
	S3	96	65722
	S4	42	43294

Reference

1. Tripathi R, Nair NN (2015) Mechanism of Meropenem Hydrolysis by New Delhi Metallo β-Lactamase. ACS Catal 5:2577–2586.
